# Graft-derived cell-free DNA, a noninvasive early rejection and graft damage marker in liver transplantation: A prospective, observational, multicenter cohort study

**DOI:** 10.1371/journal.pmed.1002286

**Published:** 2017-04-25

**Authors:** Ekkehard Schütz, Anna Fischer, Julia Beck, Markus Harden, Martina Koch, Tilo Wuensch, Martin Stockmann, Björn Nashan, Otto Kollmar, Johannes Matthaei, Philipp Kanzow, Philip D. Walson, Jürgen Brockmöller, Michael Oellerich

**Affiliations:** 1 Chronix Biomedical, Göttingen, Germany; 2 Department of Clinical Pharmacology, University Medical Center Göttingen, Göttingen, Germany; 3 Department of Medical Statistics, University Medical Center Göttingen, Göttingen, Germany; 4 Department of Hepatobiliary Surgery and Transplantation, University Medical Center Hamburg-Eppendorf, Hamburg, Germany; 5 Department of Surgery, Charité–Universitätsmedizin Berlin, Berlin, Germany; 6 Department of General, Visceral and Pediatric Surgery, University Medical Center Göttingen, Göttingen, Germany; University of Texas Southwestern Medical Center, UNITED STATES

## Abstract

**Background:**

Graft-derived cell-free DNA (GcfDNA), which is released into the blood stream by necrotic and apoptotic cells, is a promising noninvasive organ integrity biomarker. In liver transplantation (LTx), neither conventional liver function tests (LTFs) nor immunosuppressive drug monitoring are very effective for rejection monitoring. We therefore hypothesized that the quantitative measurement of donor-derived cell-free DNA (cfDNA) would have independent value for the assessment of graft integrity, including damage from acute rejection.

**Methods and findings:**

Traditional LFTs were performed and plasma GcfDNA was monitored in 115 adults post-LTx at three German transplant centers as part of a prospective, observational, multicenter cohort trial. GcfDNA percentage (graft cfDNA/total cfDNA) was measured using droplet digital PCR (ddPCR), based on a limited number of predefined single nucleotide polymorphisms, enabling same-day turn-around. The same method was used to quantify blood microchimerism. GcfDNA was increased >50% on day 1 post-LTx, presumably from ischemia/reperfusion damage, but rapidly declined in patients without graft injury within 7 to 10 d to a median <10%, where it remained for the 1-y observation period. Of 115 patients, 107 provided samples that met preestablished criteria. In 31 samples taken from 17 patients during biopsy-proven acute rejection episodes, the percentage of GcfDNA was elevated substantially (median 29.6%, 95% CI 23.6%–41.0%) compared with that in 282 samples from 88 patients during stable periods (median 3.3%, 95% CI 2.9%–3.7%; *p <* 0.001). Only slightly higher values (median 5.9%, 95% CI 4.4%–10.3%) were found in 68 samples from 17 hepatitis C virus (HCV)–positive, rejection-free patients. LFTs had low overall correlations (*r* = 0.28–0.62) with GcfDNA and showed greater overlap between patient subgroups, especially between acute rejection and HCV+ patients. Multivariable logistic regression modeling demonstrated that GcfDNA provided additional LFT-independent information on graft integrity. Diagnostic sensitivity and specificity were 90.3% (95% CI 74.2%–98.0%) and 92.9% (95% CI 89.3%–95.6%), respectively, for GcfDNA at a threshold value of 10%. The area under the receiver operator characteristic curve was higher for GcfDNA (97.1%, 95% CI 93.4%–100%) than for same-day conventional LFTs (AST: 95.7%; ALT: 95.2%; γ-GT: 94.5%; bilirubin: 82.6%). An evaluation of microchimerism revealed that the maximum donor DNA in circulating white blood cells was only 0.068%. GcfDNA percentage can be influenced by major changes in host cfDNA (e.g., due to leukopenia or leukocytosis). One limitation of our study is that exact time-matched GcfDNA and LFT samples were not available for all patient visits.

**Conclusions:**

In this study, determination of GcfDNA in plasma by ddPCR allowed for earlier and more sensitive discrimination of acute rejection in LTx patients as compared with conventional LFTs. Potential blood microchimerism was quantitatively low and had no significant influence on GcfDNA value. Further research, which should ideally include protocol biopsies, will be needed to establish the practical value of GcfDNA measurements in the management of LTx patients.

## Abstract 2

Michael Oellerich and colleagues present an approach whereby graft cell free DNA can be used for early detection of liver transplant rejection.

## Author summary

**Why was this study done?:**

- Noninvasive monitoring of graft injury after liver transplantation is needed because of the reported limitations of traditional methods used to assess liver damage and to detect rejection.
- Organ transplants are also genome transplants and therefore offer the opportunity to use graft-derived cell-free DNA (GcfDNA) as a marker of transplant organ integrity.
- GcfDNA is released into the blood circulation when cells of the transplanted organ die.

**What did the researchers do and find?:**

- In a prospective, observational, multicenter cohort study, GcfDNA was shown to be superior to traditional liver function tests (LFTs) and detected rejection as early as 8 to 15 days prior to biopsy-proven rejection.
- GcfDNA provided additional, LFT-independent information on graft rejection.

**What do these findings mean?:**

- GcfDNA shows superior and earlier discrimination of liver transplant patients with acute rejection, compared to conventional LFTs, and could improve the outcome of antirejection treatment.
- The implementation of this test in clinical practice is subject to further clinical studies.

## Introduction

Noninvasive monitoring of graft integrity after liver transplantation (LTx) is needed because of the imprecision and other limitations of traditional methods used to assess liver damage and detect rejection [1]. LTx patients must be continuously monitored for rejection episodes that—if detected—require immunosuppressant drug (ISD) adjustments. A major limitation of standard of care management is that, currently, suspected rejection episodes can be confirmed only by invasive biopsy. However, using serial biopsies to repeatedly assess graft integrity—to adjust ISD treatment and thereby individualize treatment—is often clinically impossible as well as impractical, cost-prohibitive, and a major burden for patients. Biopsies also have limited sensitivity and specificity as well as turn-around times that limit their usefulness for making rapid ISD dosing decisions. A number of conventional liver function tests (LFTs) are routinely used to assess graft function, but they are not diagnostic for assessing acute cellular or antibody-mediated rejection after LTx [2,3].

Therapeutic drug monitoring of ISDs is helpful for making some ISD dosing decisions, but it is more useful to prevent toxicity than to predict the efficacy of immunosuppressive treatment for an individual graft recipient [4–6].

Therefore, new, noninvasive biomarkers are needed that can be used to monitor graft integrity, to rapidly and reliably detect rejection, and to both minimize and individualize (i.e., “personalize”) ISD therapy. Biomarkers are needed that are practical, are cost-effective, can be used repeatedly, have rapid (same-day) turn-around time, and can be used to diagnose or predict graft damaging complications at their earliest stages. Such biomarkers could be used in real time to assess individual minimum necessary ISD exposure after transplantation. The early detection of silent graft injury (which can lead to acute rejection or chronic allograft dysfunction) would allow for earlier intervention, which is of particular importance for long-term graft survival. The need for new biomarkers is best demonstrated by the fact that despite the use of traditional monitoring methods transplant patients still often suffer from both organ rejection and ISD toxicity. Acute rejection of liver transplants within 1 y is 11.5% (age-dependent range 9.4%–20.5%) [7]. The final goal of any useful biomarker is the improvement of long-term patient and graft outcomes, by avoiding full-blown rejections as well as ISD overtreatment.

Graft-derived cell-free DNA (GcfDNA) has been shown to be a promising new biomarker for the detection of graft injury [8–10]. The fact that organ transplants are also genome transplants provides the possibility of repeated, noninvasive (i.e., “liquid biopsy”) monitoring for allograft injury through measurement of GcfDNA [11–13]. One of the earliest GcfDNA studies concluded that plasma donor DNA is a cell death marker, released from necrotic or apoptotic cells in the transplant organ, and may therefore be useful as a marker for rejection [14]. At the moment, GcfDNA can be measured either by shotgun sequencing [11,13,15] or by droplet digital PCR (ddPCR) [9] and expressed either as GcfDNA percentage (graft cfDNA/total cfDNA) or by absolute quantification in copies/milliliter [16]. Other methods based on the principle of using preselected single nucleotide polymorphisms (SNPs) [9], but using high-throughput sequencing for readout, have also been recently reported [17–19]. As sequencing is expensive, more time-consuming, and not practical in the routine hospital setting, ddPCR is superior for the rapid and cost-effective quantification of donor DNA in the circulation of transplant recipients. GcfDNA determination using ddPCR is cost-effective and, after the initial test, can provide results on the same day.

In a prospective, observational, multicenter trial, a ddPCR method for the determination of GcfDNA was evaluated as a marker of graft injury in adult LTx patients and was compared to traditional LFTs for this use.

## Methods

### Patients

**Fig 1:**
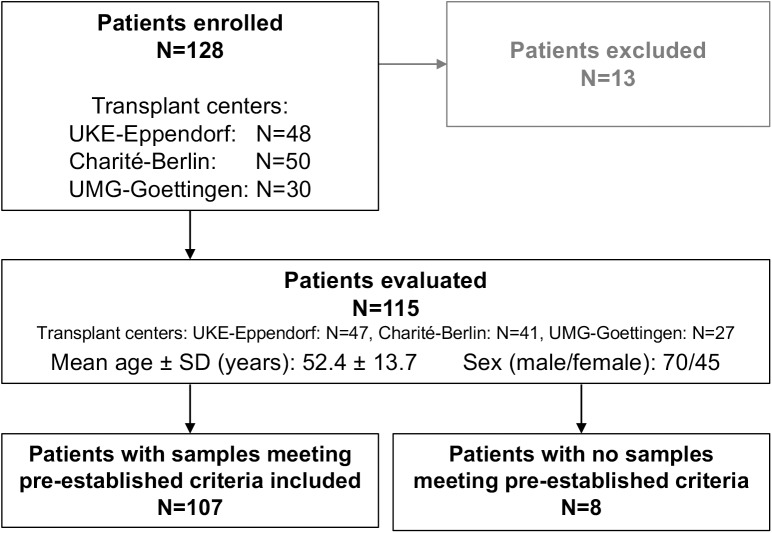
Flowchart displaying patient enrollment. Charité-Berlin, Charité–Universitätsmedizin Berlin; SD, standard deviation; UKE-Eppendorf, University Medical Center Hamburg-Eppendorf; UMG-Goettingen, University Medical Center Göttingen.

Between October 2012 and September 2015, a total of 128 adult LTx patients receiving a liver graft were enrolled in a prospective, multicenter, non-interventional, observational trial comparing GcfDNA percentage with clinical events, conventional LFTs, ISD therapeutic drug monitoring, and biopsy results for detecting graft injury (Fig 1). There were 13 patients excluded as described in S1 Table.

Demographics of all evaluated patients are shown in Table 1. Serial determinations as defined in the section about experimental monitoring were made for up to 1 y post-transplant in the 115 liver recipients who were recruited at three major German transplant centers (Charité–Universitätsmedizin Berlin, University Medical Center Hamburg-Eppendorf [UKE], and University Medical Center Göttingen [UMG]) (Fig 1). A total of 107 patients were included according to preestablished criteria described below. All patients had been treated in the respective center’s regular transplant program. The basic immunosuppressive regimens used at all three centers typically consisted of induction therapy with basiliximab or antithymocyte globulin (ATG), corticosteroids (variable withdrawal post-transplant), and calcineurin inhibitors (cyclosporine or tacrolimus). Additional administration of mycophenolate mofetil (*N =* 78), everolimus (*N =* 42), or azathioprine (*N =* 7) was documented. ISD dosing was adapted to maintain locally determined target trough ISD concentrations in accordance with the locally used therapeutic ranges.

**Table 1:** Demographics and baseline characteristics.

Characteristic	Overall (*N =* 115)		Gender				Sample subgroups (*N =* 107)*, *N* or mean ± SD		
	*N* total or *N* de novo, *N* re-transplant	Percent or mean ± SD	Female (*N =* 45)		Male (*N =* 70)		Stable	HCV+	bpar
			*N*	Percent or mean ± SD	*N*	Percent or mean ± SD			
**Patients evaluated**	115	100.0%	45	100.0%	70	100.0%	88*	17*	17*
**Age (years)**	115	52.4 ± 13.7	45	49.8 ± 15.1	70	54.1 ± 12.5	51.3 ± 14.5	58.6 ± 6.3	46.2 ± 16.2
**Race**									
Caucasian	113	98.3%	44	97.8%	69	98.6%	86	17	17
Black	1	0.9%	1	2.2%	0	0.0%	1	0	0
Oriental	1	0.9%	0	0.0%	1	1.4%	1	0	0
**Indication for LTx**									
Cirrhosis	31, 4	30.4%	13	37.1%	22	62.9%	23	9	3
Cirrhosis with HCC	25, 1	22.6%	6	23.1%	20	76.9%	18	6	3
PSC	16, 2	15.7%	8	44.4%	10	55.6%	17	0	4
AIH, cirrhosis	4, 0	3.5%	2	50.0%	2	50.0%	4	0	0
Acute liver failure	5, 0	4.3%	4	80.0%	1	20.0%	4	0	1
HCC	3, 0	2.6%	1	33.3%	2	66.7%	2	1	0
PCLD	3, 0	2.6%	3	100.0%	0	0.0%	2	0	1
SSC	3, 2	4.3%	1	20.0%	4	80.0%	4	0	2
Hemochromatosis	2, 0	1.7%	0	0.0%	2	100.0%	2	0	0
Primary nonfunction	0, 3	2.6%	3	100.0%	0	0.0%	3	0	0
Chronic liver failure	0, 3	2.6%	0	0.0%	3	100.0%	3	0	0
Chronic rejection	0, 2	1.7%	1	50.0%	1	50.0%	1	0	1
ADPKD with liver cysts	1, 0	0.9%	1	100.0%	0	0.0%	1	0	1
EHE	1, 0	0.9%	1	100.0%	0	0.0%	1	0	1
Iatrogenic liver necrosis	1, 0	0.9%	0	0.0%	1	100.0%	1	0	0
Liver adenoma	1, 0	0.9%	1	100.0%	0	0.0%	1	0	0
Bile duct necrosis	0, 1	0.9%	0	0.0%	1	100.0%	0	1	0
Ischemic graft failure	0, 1	0.9%	0	0.0%	1	100.0%	1	0	0
MELD score**	120***	20.0 ± 10.4	45	22.3 ± 11.0	70	18.4 ±9.7	20.1 ± 10.3	18.6 ± 11.7	19.4 ± 9.7
**Age of donor (years)**	120***	53.4 ± 16.8	45	47.2 ± 16.7	70	57.7 ±15.5	54.0 ± 16.6	53.3 ± 17.8	45.9 ± 13.5
**CIT (hours)**	120***	10.0 ± 2.5	45	9.5 ± 2.1	70	10.4 ± 2.6	9.8 ± 2.3	10.3 ± 1.6	10.1 ± 2.5
**Split liver**	4, 1 of 120***	4.2%	3	6.7%	2	2.9%	4	1	1

*The sample subgroups consisted of a total of 107 LTx patients. In all, 88 HCV− patients had samples collected during stable phase. However, 13 of these patients also had samples collected during the bpar interval, as did two of the 17 HCV+ patients.

**MELD score is a disease severity index to help prioritize allocation of organs for transplant based on serum creatinine, serum bilirubin, and international normalized ratio of prothrombin time.

***120 transplantations were performed in the 115 evaluated patients (Fig 1). The five additional transplantations were the result of one patient who was re-transplanted once and two patients who each received two re-transplantations.

ADPKD, autosomal dominant polycystic kidney disease; AIH, autoimmune hepatitis; bpar, biopsy-proven acute rejection; CIT, cold ischemia time; EHE, epithelioid hemangioendothelioma; HCC, hepatocellular carcinoma; HCV, hepatitis C virus; LTx, liver transplantation; MELD, Model for End-Stage Liver Disease; PCLD, polycystic liver disease; PSC, primary sclerosing cholangitis; SD, standard deviation; SSC, secondary sclerosing cholangitis.

Treatment of biopsy-proven or clinically suspected acute rejection was by steroids (250 mg/d–1 g/d over up to 3 d) and/or increased dosage or switches of standard immunosuppression.

During the first year post-LTx, nine out of 115 evaluated patients died (S2 Table) and three patients were re-transplanted, of whom two were re-transplanted twice during the study.

The study protocol conformed to the ethical principles of the 1975 Declaration of Helsinki as reflected in the a priori approval by the ethics committees of all three centers (approval numbers: UMG: 17/7/12; UKE: PV4125; Charité–Universitätsmedizin Berlin: EA1/299/12), and written, informed consent was obtained from each patient.

Treating physicians were not given GcfDNA results during this prospective, non-intervention study, and the laboratory performing the ddPCR had no access to clinical data until the study was completed. Unblinding was done after enrolled patients were at least 1 y post-transplant.

### Experimental monitoring

Repeated GcfDNA determinations were scheduled to be performed in the study patients at specific per-protocol postoperative visits (V1: 0–10 d; V2: 11–30 d; V3: 1–2 mo; V4: 2–4 mo; V5: 4–8 mo; V6: 8–10 mo; and V7: 10–14 mo) as well as whenever rejection was suspected clinically (S3 Text). Missing a per-protocol visit (V1–V7) was not an exclusion criterion. An average of 4.9 of the seven per-protocol visits were recorded for the 115 evaluated patients (Fig 1). In order to assess early ischemia/reperfusion damage, additional GcfDNA testing was performed at closer intervals during the first 2 wk in a subset of 24 patients from UMG.

Blood (10 ml) was drawn into special tubes (Cell-Free DNA BCT, Streck) for GcfDNA determination. The stability of GcfDNA in whole blood is at least 7 d in these tubes at 6–37°C [20]. After plasma separation, which was performed within this time frame, plasma samples were stored at −20°C prior to DNA extraction. EDTA blood was used to measure cyclosporine, tacrolimus, and everolimus levels, and EDTA plasma to measure mycophenolic acid trough levels, by liquid chromatography tandem mass spectrometry (LC-MS/MS). LFTs and ISD concentrations were measured as per local laboratory routine analyses, and all biopsies were performed, analyzed, and reported as per standard of care at the three different transplant centers. The indication for biopsy was based on elevated LFTs and further criteria such as prolonged prothrombin time, reduced concentration of coagulation factor V, and suspicious ultrasound. Protocol biopsies are generally not performed in the participating centers because of risk/benefit considerations.

GcfDNA was measured by ddPCR as described previously, where all PCR conditions, primers, and probes are documented [9]. Briefly, cfDNA was isolated from plasma and subjected to allele-specific ddPCR of SNP loci with known high population minor allelic frequency, selected from a predefined set of over 40 different SNP loci. For each patient, four to five informative SNP assays—for which the recipient showed homozygosity and the donor differed—were selected from an individual prescreen of the total set and used to quantify the GcfDNA fractional abundance. Prescreening involved (i) genotyping the 40 SNPs using the recipient’s white blood cell (WBC) DNA in real-time PCR format and (ii) testing all SNPs homozygous in the recipient for the presence of at least one heterologous allele in pre-amplified cell-free DNA (cfDNA) in ddPCR format, where homozygous graft SNPs can be distinguished from heterozygous by their double percentage. The assay selection procedure was applied only once for each patient, and all subsequent samples were tested only with the patient’s personal informative assay set. Final GcfDNA percentages were obtained by summing the fractional abundances of the 4–5 informative assays, where heterozygote SNP values were multiplied by two and the result divided by the number of used assays. Based on population statistics, only one in about 35 million random donor–recipient combinations would not reach the number of four informative loci for GcfDNA quantification when 40 loci, selected as above, are prescreened. In living related transplantation, parent–offspring combinations would lead to one in about 1,000, and siblings to one in about 125,000, untestable combinations; use of this technique is not applicable for transplantation between identical twins. All testing was done using a QX200 Droplet Digital PCR System (Bio-Rad).

Sample source was masked by assigning a unique study number to each patient, which was used for sample tracking throughout the study.

### Criteria for inclusion in acute rejection versus stable patient subgroups

Percutaneous liver biopsies were performed in cases of clinically suspected acute rejection and were evaluated by experienced pathologists at the respective treating transplant center. Because in the clinical setting GcfDNA values were not obtained exactly at the time of biopsy, we selected as results representing rejection only those samples collected during a time interval from ≤6 d before to 1 d after the time of biopsy, starting at day 14 post-LTx. No samples were drawn within 12 h after a biopsy. Because the half-life of GcfDNA is <1.5 h, this 12-h delay is sufficient to eliminate the possibility of the biopsy itself elevating GcfDNA and confounding the results. Six samples from five patients (one of whom had two biopsies) were drawn the day after biopsy. In one of those patients, GcfDNA values were available 1 d before and 1 d after biopsy, with GcfDNA values of 39.6% and 45.1%, respectively. In a further patient with biopsy-confirmed non-rejection, the GcfDNA value after 1 d was <10%. These data confirm that the chosen time interval is appropriate. Also, since high-dose bolus steroid treatment of acute rejection results in rapid decreases in GcfDNA [21], samples collected during the above-mentioned interval (≤6 d before to 1 d after the time of biopsy) were included only if no steroid boluses were given during this period of time. In this study, samples during biopsy-proven acute rejection (bpar) were collected during a time interval from ≤ 6 d before to 1 d after the time of biopsy in 17 patients and were included for the analytical comparisons in this report (Fig 1; Table 1). No occurrences of bpar were seen later than 6 mo after surgery.

Samples categorized as representing “stable periods” had to fulfill the following criteria based on a review of the patient records: sample collected at least 14 d post-LTx, absence of rejection (i.e., no bpar) in the 15 d prior to sampling, and no documented complication at time of protocol-based blood sampling. Exclusion criteria were as follows: infection (viral or bacterial) of the liver or bile ducts, plasmapheresis or MARS (i.e., liver dialysis) therapy, invasive interventions within −7/+3 d of sample collection, laparotomy within 5 d after sample collection, ≤7 d after any steroid bolus, bile duct leakage or necrosis, hepatocellular carcinoma, ischemia due to reduced perfusion within 5 d after sample collection, hematoma or cysts in the liver, abdominal negative pressure therapy, cholestasis, sepsis, transplant failure, pregnancy, or additional kidney transplantation.

Out of the 115 patients evaluated, 107 patients had samples that met all preestablished inclusion criteria (Fig 1; Table 1). Out of these, there were samples collected during a stable period from 88 patients, hereafter referred to as “stable patients.” Results for these samples were compared to results from 17 patients with hepatitis C virus (HCV) recurrences and positive HCV RNA PCR tests but without evidence of rejection, and to samples drawn during bpar episodes in 17 patients. Details of the eight patients whose samples all failed to meet the preestablished inclusion criteria are given in S3 Table.

To assess microchimerism, DNA extracted from theWBCs of 12 LTx patients was used for ddPCR with four SNP assays selected for the donor–recipient pair as described above. The donor-derived fraction of the WBC DNA was quantified in this subset of 12 patients, selected because they had a GcfDNA percentage > 10% during a non-rejection period. Each sample was compared to (i) a second sample from the same patient at a time when GcfDNA percentage was less than 10% and (ii) a sample from a different patient drawn the same post-operative week. For each sample, a total of 39,200 positive droplets (sum of both alleles) were generated, which translates into the ability to detect 0.0075% donor WBCs with 95% certainty.

### Statistical analysis

All statistical analyses were performed using SAS version 9.4. Continuous data are presented by median and 95% confidence interval or mean and standard deviation, whereas frequencies are reported as proportions and exact 95% Clopper–Pearson confidence intervals. Laboratory results are graphically expressed as box plots showing the 5th, 25th, 50th, 75th, and 95th percentiles. GcfDNA and LFTs (aspartate aminotransferase [AST], alanine aminotransferase [ALT], γ-glutamyltransferase [γ-GT], and bilirubin) were compared between stable, HCV+, prior to bpar, and bpar subgroups, using a linear mixed effects model assuming normally distributed data and accounting for repeated measurements to test the null hypothesis of equal expectations between groups. Where there was a significant overall effect, multiple comparisons were performed. *p*-Values were adjusted based on the Bonferroni correction. *p*-Values < 0.05 were considered statistically significant. Laboratory results from patients with bpar who had adequate, repeated GcfDNA measurements were also examined to determine how long prior to a positive biopsy GcfDNA elevations were seen. Finally, all GcfDNA and LFT elevations were also compared to the presence of either clinical or biopsy-proven diagnoses of rejection in the medical records.

Spearman correlation coefficients were calculated between GcfDNA percentages and traditional LFTs (AST, ALT, γ-GT, glutamate dehydrogenase [GLDH], and bilirubin) over the observation period, including samples from three subgroups: stable, HCV+, and bpar. Since GLDH was systematically monitored in only one (UKE) of the three centers, GLDH measurements were excluded from the overall analysis, but a single-center analysis was performed to compare GcfDNA percentages against LFTs including GLDH at this center.

Receiver operator characteristic (ROC) analyses were performed to assess which laboratory marker (GcfDNA or traditional LFTs) discriminated best between bpar and stable as well as HCV+ samples. To ensure comparability of the analyses, samples were included only when results were available for all compared analytes on the same day. Additionally, also only when same-day analyses were available, a single-center analysis was performed for GLDH measurements. Threshold values were calculated either resulting from a specificity of at least 95%, for comparing stable against bpar samples, or based on the maximum Youden index, for comparing HCV+ against bpar samples. To assess whether GcfDNA provided independent value for the classification of bpar samples, multivariable logistic regression models were fitted to the data. Finally, a ROC analysis was performed that included all available stable or bpar GcfDNA percentage samples to analyze the diagnostic sensitivity and specificity at a threshold value of 10%.

## Results

### Time dependence of GcfDNA percentage in liver transplant patients without rejection, complications, or infection

**Fig 2:**
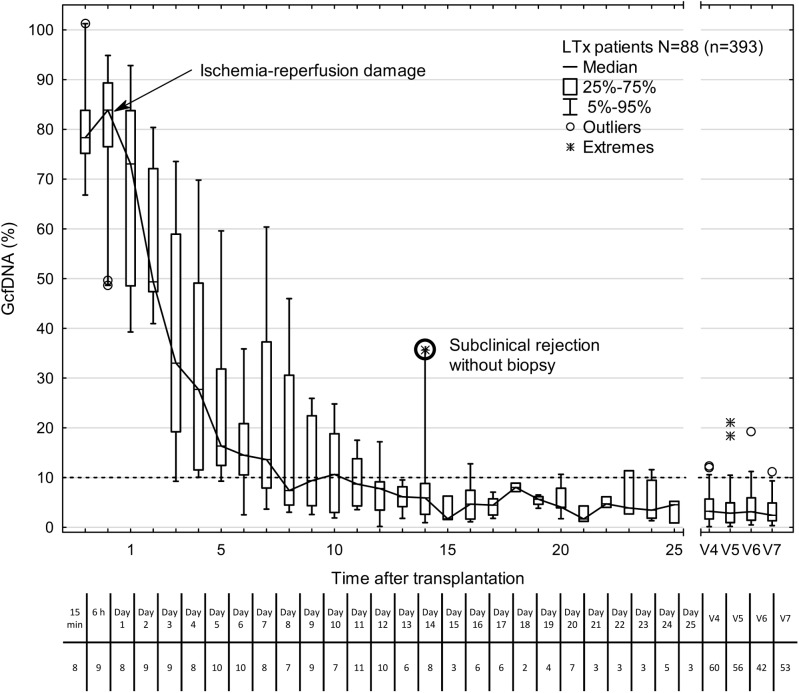
Time course of plasma GcfDNA percentage during the first year after liver transplantation in patients without rejection, active infections, or interventions. Boxes represent median with interquartile range, with whiskers showing the 5th–95th percentile. V4, 2–4 mo; V5, 4–8 mo; V6, 8–10 mo; and V7, 10–14 mo. The number of samples is given below each time point. The bold circle represents one patient with subclinical rejection without biopsy 1 wk earlier (see text). GcfDNA, graft-derived cell-free DNA; LTx, liver transplantation.

The GcfDNA percentages were highly elevated on the first days after transplantation, most likely reflecting ischemia/reperfusion damage (Fig 2), but the median GcfDNA percentage decreased within the first 7–10 d to a level below 10% in stable patients with no signs of graft injury. GcfDNA values (*n =* 393) obtained in stable patients (*N =* 88) then remained low throughout the first month (Fig 2) as well as during the entire observation period of 1 y. An outlier (on day 14; Fig 2) is described in detail below.

### GcfDNA in stable liver transplant patients, HCV+ patients, and patients with biopsy-proven acute rejection

**Fig 3:**
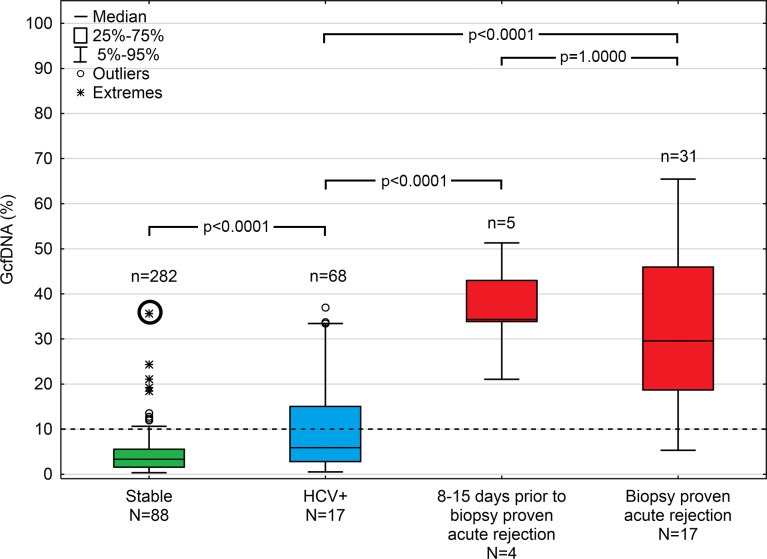
Plasma GcfDNA percentages during the first year after transplantation in stable patients and patients with either HCV or biopsy-proven acute rejection. Boxes represent median with interquartile range, with whiskers showing the 5th–95th percentile and *n*’s showing the number of contributing values. The bold circle represents the patient with subclinical rejection without biopsy 1 wk earlier (see text). GcfDNA, graft-derived cell-free DNA; HCV, hepatitis C virus.

Fig 3 summarizes GcfDNA percentages obtained during the first year from 88 LTx patients during a stable phase, as well as HCV+ patients and those with bpar episodes. Stable patients showed GcfDNA values below 10%, indicating no graft damage. Of note, elevated (19.2% to 37.5%) GcfDNA values were observed in four patients who were clinically judged to be stable despite having elevated LFTs. One of these patients, with a GcfDNA percentage of 37.5% on day 14 after LTx, had highly elevated LFTs as well, presumably due to a subclinical but controlled rejection because of insufficient (subtherapeutic) tacrolimus blood levels 1 wk earlier. In this patient, after ISD dosage adjustment, GcfDNA percentage subsequently decreased to 5.6% on post-operative day 35. The other three stable patients who had GcfDNA percentages between 19.2% and 24.3% fulfilled the criteria to be included in the stable group; however, their LFTs were slightly increased at these visits. Overall, the data confirm the previously described [9] upper GcfDNA threshold of 10% for stable LTx patients, with a median GcfDNA percentage among stable patients in our study of 3.3% (95% CI 2.9%–3.7%).

Samples (*n =* 68) from 17 HCV+ patients, as to be expected, had slightly higher and more variable GcfDNA percentage results (median 5.9%, 95% CI 4.4%–10.3%). Samples (*n =* 9) from seven patients with cholangitis and otherwise uncomplicated courses had a median GcfDNA percentage of 5.3% (range 0.8%–13.5%). In three patients with documented cholestasis and no obvious liver damage, GcfDNA percentage values were ≤ 9.9%.

In contrast, 31 samples from 17 patients with bpar had the highest GcfDNA percentage values (median 29.6%, 95% CI 23.6%–41.0%). There were two patients with positive biopsies whose GcfDNA percentage was not elevated above 10%. In one patient, raised GcfDNA percentages may have been masked by elevated levels of total cfDNA since spiking leukocyte counts were measured at the time of sampling. The other patient had a biopsy showing mild rejection (grade 1) on the same day the GcfDNA percentage was 5.3%. This patient had a moderately differentiated biopsy result (grade 2) and a GcfDNA percentage of 11.5% 1 wk prior (day 6 post-op), which was treated in the interim with a steroid bolus and ATG.

Three of the bpar episodes were diagnosed during the first post-LTx week. The respective GcfDNA percentage values were 86.1% on day 3, 82.1% on day 6, and 28.4% on day 11 (versus a median of 33%, 14.5%, and 8.7% in rejection-free patients on the respective days). Data obtained for these rejections were excluded from all further analyses.

Additionally, in four rejection episodes, samples were available before clinical suspicion of rejection. In all these cases, elevated GcfDNA percentage values (median 34.3%) were seen as early as 8–15 d before acute cellular rejection was confirmed by biopsy.

### Comparison of GcfDNA to conventional liver function tests

Compared to traditional LFTs, there was much better discrimination in GcfDNA results between patients with bpar, HCV+ patients without rejection, and stable patients. The LFTs (AST, ALT, γ-GT, and bilirubin) in such patients showed much greater overlap between subgroups (S1–S4 Figs). Additionally, significant increases in GcfDNA were seen up to 1–2 wk before bpar was diagnosed, compared to stable and HCV+ patients (Fig 3). The Spearman correlation coefficients (*r*) between GcfDNA results and conventional LFTs in stable patients, HCV+ patients, and patients with bpar episodes are shown in Table 2. Despite being statistically significant, the correlations were quite low (*r* values ranging from 0.28 to 0.62). As GLDH was systematically measured in only one of the centers, a single-center analysis for GLDH is given in S4 and S5 Tables. The correlation for GLDH (*r* = 0.49) was within the range of the other LFTs (*r* values ranging from 0.26 to 0.63).

**Table 2:** Correlation between GcfDNA percentages and conventional liver function tests in adult liver transplant patients (acute rejection, HCV+, or stable).

GcfDNA	AST	ALT	γ-GT	Bilirubin
GcfDNA percentage	0.46 (0.37–0.55)	0.62 (0.54–0.68)	0.45 (0.36–0.54)	0.28 (0.17–0.38)
*p*-Value	<0.0001	<0.0001	<0.0001	<0.0001

Spearman correlation coefficients are given with 95% CIs; 317 samples.

GcfDNA, graft-derived cell-free DNA; HCV, hepatitis C virus.

To compare the diagnostic performance of the different biomarkers, ROC curves were plotted and the area under the curve (AUC) was calculated. These analyses were performed with 232 samples from 80 stable patients and 28 samples collected during the bpar interval in 16 patients, for whom same-day data for each test were available, and the results are shown in Table 3. GcfDNA demonstrated a distinctly better identification of patients with acute rejection than did any LFT, with an AUC of 97.1% (95% CI 93.4%–100%). For the conventional LFTs, the AUC of AST was best, whereas bilirubin had the worst separation. As a more intuitive, harmonized comparison, the sensitivity for rejection detection was calculated for each biomarker at the point of 95% specificity (Table 3).

**Table 3:** Diagnostic sensitivity and respective thresholds at 95% diagnostic specificity obtained from receiver operator characteristic curves in rejection versus stable period samples.

Measure	*n*	AUC		Sensitivity (*n =* 28)		Specificity (*n =* 232)		Threshold at 95% specificity
		Percent	95% CI	Percent	95% CI	Percent	95% CI	
GcfDNA percentage	260	97.1	93.4–100.0	89.3	71.8–97.7	95.7	92.2–97.9	11.2%
AST	260	95.7	90.7–100.0	82.1	63.1–93.9	95.7	92.2–97.9	56 U/l
ALT	260	95.2	90.1–100.0	85.7	67.3–96.0	95.7	92.2–97.9	66 U/l
γ-GT	260	94.5	91.4–97.7	71.4	51.3–86.8	95.7	92.2–97.9	387 U/l
Bilirubin	260	82.6	73.6–91.6	50.0	30.6–69.4	95.7	92.2–97.9	30.8 μmol/l

AUC, area under the curve; GcfDNA, graft-derived cell-free DNA.

A similar ROC analysis was performed for 57 same-day samples from 16 HCV+ patients compared to 28 same-day measured values from 16 patients with bpar. GcfDNA percentage again performed best, followed by ALT (Table 4). As would be expected, the thresholds were higher for most parameters than for the comparisons between samples from stable, HCV− patients and bpar patients (Table 3).

**Table 4:** Youden-index-based diagnostic sensitivity and specificity obtained from receiver operator characteristic curves in rejection versus HCV+ samples.

Measure	*n*	AUC		Sensitivity (*n =* 28)		Specificity (*n =* 57)		Threshold at maximum YI
		Percent	95% CI	Percent	95% CI	Percent	95% CI	
GcfDNA	85	88.2	80.6–95.9	75	55.1–89.3	84.2	72.1–92.5	23.6%
AST	85	76.3	65.4–87.2	71.4	51.3–86.8	75.4	62.2–85.9	82 U/l
ALT	85	83	72.8–93.2	64.3	44.1–81.4	89.5	78.5–96.0	132 U/l
γ-GT	85	75.3	65.1–85.5	67.9	47.6–84.1	71.9	58.5–83.0	430 U/l
Bilirubin	85	68.1	55.6–80.6	64.3	44.1–81.4	73.7	60.3–84.5	22.2 μmol/l

AUC, area under the curve; GcfDNA, graft-derived cell-free DNA; YI, Youden index.

**Fig 4:**
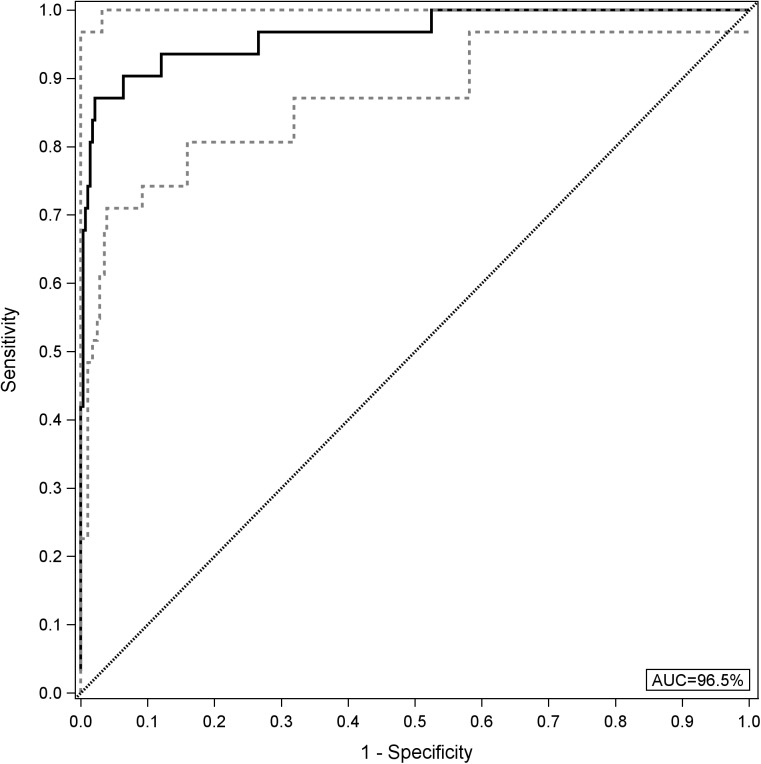
Receiver operator characteristic curve for GcfDNA. All graft-derived cell-free DNA percentage values were considered (*n =* 282 samples from stable periods and *n =* 31 samples during biopsy-proven acute rejection). The upper and lower limits of the 95% CI are shown as dashed lines. AUC, area under the curve.

An analysis using all GcfDNA values obtained for stable periods or bpar episodes (*n =* 313) at a cutoff value of 10% GcfDNA yielded a diagnostic sensitivity of 90.3% (95% CI 74.2%–98.0%) and a specificity of 92.9% (95% CI 89.3%–95.6%). The respective ROC curve is given in Fig 4. The AUC was 96.5% (95% CI 92.7%–100%).

In order to assess whether GcfDNA percentage has independent value for distinguishing rejection episodes from stable courses, multivariable logistic regression results for LFTs with and without GcfDNA were calculated. As shown in Table 5, GcfDNA percentage showed an effect independent of the LFTs, of which AST was the best parameter. Table 5 shows the detailed results for the model including GcfDNA.

**Table 5:** Multivariable logistic regression results for liver function tests and GcfDNA.

Parameter	DF	Estimate	Standard error	Wald Chi-square	*p*-Value
Intercept^1^	1	−6.6435	1.0510	39.9597	<0.0001
GcfDNA percentage	1	0.1800	0.0515	12.2256	0.0005
AST	1	0.0559	0.0266	4.4182	0.0356
ALT	1	−0.0073	0.0142	0.2664	0.6057
γ-GT	1	0.00145	0.0011	1.8124	0.1782
Bilirubin	1	−0.4537	0.4134	1.2044	0.2724

^1^Constant term in linear regression analysis (value at which the fitted line crosses the *y*-axis).

DF, degrees of freedom; GcfDNA, graft-derived cell-free DNA.

The global Chi-square of the likelihood ratio for the ROC model for LFTs and GcfDNA percentage was 138.9. (degrees of freedom = 5, −log *p =* 27.5), whereas the value without GcfDNA percentage was 122.5 (degrees of freedom = 4, −log *p =* 24.8). In the latter analysis, again only AST had a significant influence. Results suggest a high interdependence of the conventional LFTs. Additional statistics for these comparisons are given in S6 and S7 Tables.

### Investigation into blood microchimerism

The overall average percentage of donor DNA in circulating WBCs was 0.017% in the tested 12 LTx patients, with a maximum of 0.068%. Three patients had no detectable microchimerism. The comparison between high and low GcfDNA sample pairs (from the same patient) showed no significant difference (*p =* 0.22), as was also true for the comparison to other patient samples collected on the same post-operative day (*p =* 0.9). There was also no difference between samples drawn within the first 2 mo after engraftment compared to samples from later than 3 mo (*p =* 0.39).

The overall very low level of donor DNA detected in circulating WBCs collected in parallel with cfDNA essentially excludes microchimerism as a confounding source of the GcfDNA elevations noted.

## Discussion

The results from this prospective, observational, multicenter study confirm previous preliminary reports from our group, that plasma GcfDNA measured in LTx patients is a very useful marker of graft integrity, identifying patients with acute rejection better than conventional LFTs and with a clinically acceptable turn-around time. Changes observed in AST, ALT, γ-GT, and bilirubin showed more overlap between patients with bpar, those with HCV infection, and stable patients. The results of ROC analysis indicate a higher diagnostic sensitivity at 95% specificity for GcfDNA percentage compared to conventional LFTs.

Highly elevated GcfDNA percentage was consistently demonstrated immediately after engrafting, followed by a steady decrease to reach a plateau after 1 wk at approximately 10% of total circulating cfDNA in the 88 stable phase patients (Fig 2). The initial elevation is presumably a result of ischemia/reperfusion damage, and the decline curve might therefore be a more direct indication of this well-known damage to cadaveric donor grafts, since GcfDNA release is due to necrotic and apoptotic events in the graft and therefore should reflect their magnitude.

Even during this early phase, when the clinical course can be difficult to assess, values greater than 2.5 times the median value for a given post-operative day suggest rejection. However, elevated values have also been seen in some cases with other severe graft injuries (S3 Table). These findings are in line with earlier observations showing that a sustained and rapid decline in GcfDNA during this early phase can be a good prognostic indicator even in marginal donor grafts [22]. The serial view of patients’ data is of additional value, since any increase during this early phase should also alert the clinician to a graft injury complicating the peri-operative course. After the first 10 d post-LTx, GcfDNA percentage remained stable (median < 10%) in the absence of rejection or other causes of graft damage during the first year after transplantation, and this level could be used as a threshold.

Cross-sectional evaluation of the data after the initial Tx phase allows for estimation of the usefulness of GcfDNA percentage in the clinical setting for liver recipients. The data collected for the first year show the overall superiority of GcfDNA quantification compared to conventional LFTs, as shown particularly in Tables 3–5. In an independent discrepancy analysis done after unblinding, there was one patient with elevated GcfDNA percentage who erroneously had no rejection reported on the case report form, as well as two patients with rejection diagnoses that did not hold true on re-evaluation. These analyses were very reassuring that GcfDNA percentage can serve as a very precise diagnostic biomarker.

The overall superiority of GcfDNA as a biomarker was also seen in the ROC comparisons with conventional LFTs, where GcfDNA consistently demonstrated the highest AUC of the ROC curves (Tables 3 and 4). The results also demonstrate that GcfDNA provides additional independent information on graft integrity (Table 5). The need to better distinguish between HCV recurrence and acute rejection has been emphasized [1]. One major difference in the biology underlying GcfDNA and LFTs is the fact that GcfDNA percentage is based on the precise number of DNA copies per cell in a diploid mammalian organism. The DNA shed from an organ is directly correlated with the number of dying cells, with two copies per one cell. The amounts of all other markers used are dependent on a plethora of biochemical pathways and obstacles, such as production of the marker (e.g., bilirubin), RNA expression and protein synthesis (e.g., enzymes), and intracellular partitioning leading to different leakage (e.g., AST >> GLDH). This is not the case for GcfDNA. Since a cell without DNA is not viable, whatever DNA is detected in the circulation reflects cell death in the graft.

These considerations and the results presented suggest that GcfDNA can be used as a “liquid biopsy” to directly and repeatedly interrogate graft integrity. The direct assessment of organ integrity could lead to earlier detection of acute rejection that could help to provide more timely, effective, and individualized therapy. This would be especially useful during ISD minimization attempts [23] and when assessing the efficacy of therapies such as steroid boluses or changes in ISD dosing. Here the very short cfDNA half-life of <1.5 h [9,24] plays a major role, since it enables an almost real-time view of the graft. Aminotransferases, for example, have a much longer half-life (i.e., 17 h for AST and 47 h for ALT) [25]. Biopsies, on the other hand, are invasive and expensive, have major risks, have relatively long turn-around times, and are subject to both sampling errors and subjective interpretations [26]. GcfDNA might also be useful to guide initial ISD regimen selection in patients who need more aggressive therapy because of an increased rejection risk or patients who need less aggressive dosing because of the presence of infections or an increased risk of infections or other side effects of ISD therapy.

Also, treatment of acute rejection with steroids results in a rapid decline of GcfDNA, allowing for an early evaluation of efficacy [21,22]. Failure of elevated GcfDNA values to fall after steroid boluses was associated with non-rejection-related causes of graft damage [22] or steroid-resistant rejection [21]. While specific, controlled trials are needed to test the hypothesis, it is likely from the cases seen so far that changes in GcfDNA in response to bolus steroids can be used to confirm steroid-responsive rejection versus either nonresponsive rejection or other non-rejection-related causes of graft damage.

ISD switches are often performed to avoid or minimize ISD toxicity. Under-immunosuppression may occur during such switches and can result in acute rejection [22]. Repeated GcfDNA measurements during such ISD switches may be especially useful to monitor graft integrity and detect subclinical or acute rejection. ISD concentration measurements are often of limited use during such switches.

There are a few practical limitations to the GcfDNA methods used in this study. For routine application, there is a need for access to ddPCR equipment and the qualified personnel necessary to generate reliable results and to interpret them. The cost of an individual test is moderate and much less than that of, e.g., massive sequencing. The GcfDNA percentage reflects the relative percentage of graft cfDNA in plasma (graft cfDNA/total cfDNA). In an attempt to determine the organ source of cfDNA by epigenetic modeling [27], it was found that the majority is coming from circulating WBCs, and about 10% from the liver, which is in line with the upper threshold for GcfDNA percentage that was established for stable LTx patients. GcfDNA percentage can be affected by changes in both graft and recipient cfDNA. Leukopenia as well as leukocytosis may also alter the GcfDNA percent. It is well known from cfDNA-based noninvasive prenatal testing that the body mass index (BMI) can also play a confounding role [28], e.g., higher values of cfDNA for patients with extremely low BMI and lower values for patients with high BMI. For such cases, the percentage values can be expressed as copies/milliliter plasma by adding a total cfDNA quantification [12,16]. Overall, for LTx this does not seem to be necessary, most likely because of the high percentage of graft DNA. After transplantation of other organs such as heart and kidney, with lower GcfDNA percentages [9,13], the assay can be adapted easily to the lower expected amount of graft-derived copies.

In three patients who met the stable period criteria (Fig 3), elevated GcfDNA values (range 18.4%–24.3%) were observed. These patients also had mildly elevated LFTs, below their respective rejection thresholds, as shown in Table 3. GcfDNA values suggest more severe damage was present than suspected from the LFTs alone. In contrast, there were two patients with acute rejection diagnoses but without elevated GcfDNA (3.1% and 5.3%). In both patients, LFTs were below rejection thresholds as well (Table 3), except for one γ-GT value of 734 U/l. Both patients were receiving antirejection therapy, and samples were collected during the decay phase after successful treatment of bpar. The short half-life of GcfDNA leads to lower values at these sampling time points, and the low-grade lymphocyte infiltration still seen in the control biopsies paralleled the respective GcfDNA values.

The results of this prospective, observational, multicenter study suggest that GcfDNA determination in plasma using ddPCR allows for a more sensitive and 1–2 wk earlier discrimination of LTx patients with acute rejection, compared to conventional LFTs. GcfDNA measurement may be helpful in achieving more effective personalized immunosuppression. Further research, which ideally should include protocol biopsies, will be needed to establish the practical value of GcfDNA measurements in the management of LTx patients.

## Supporting information

S1 Data Full dataset underlying the reported findings. (XLSX)

S1 Fig Plasma AST values obtained during the first year after transplantation in patients who were stable, were HCV+, or had biopsy-proven acute rejection. Boxes represent median with interquartile range, with whiskers showing the 5th–95th percentile and *n*’s showing the number of contributing values. *N* is the number of patients.
(TIF)

S2 Fig Plasma ALT values obtained during the first year after transplantation in LTx patients who were either stable, were HCV+, or had biopsy-proven acute rejection. Boxes represent median with interquartile range, with whiskers showing the 5th–95th percentile and *n*’s showing the number of contributing values. *N* is the number of patients.
(TIF)

S3 Fig Plasma γ-GT values obtained during the first year after transplantation in patients who were stable, were HCV+, or had biopsy-proven acute rejection. Boxes represent median with interquartile range, with whiskers showing the 5th–95th percentile and *n*’s showing the number of contributing values. *N* is the number of patients.
(TIF)

S4 Fig Plasma bilirubin values obtained during the first year after transplantation in patients who stable, were HCV+, or had biopsy-proven acute rejection. Boxes represent median with interquartile range, with whiskers showing the 5th–95th percentile and *n*’s showing the number of contributing values. *N* is the number of patients.
(TIF)

S1 STROBE Checklist STROBE statement. (DOC)

S1 Table Details for excluded patients. (DOCX)

S2 Table Evaluated patients who died within the first year after liver transplantation. (DOCX)

S3 Table Patients with complicated courses and who failed to meet preestablished criteria. (DOCX)

S4 Table Correlation between GcfDNA and conventional liver function tests including GLDH in adult liver transplant patients from University Medical Center Hamburg-Eppendorf. (DOCX)

S5 Table Diagnostic sensitivity at a 95% diagnostic specificity obtained from receiver operator characteristic curves in rejection versus stable period samples after day 14 in data from University Medical Center Hamburg-Eppendorf. (DOCX)

S6 Table Additional multivariable logistic regression results for liver function tests and GcfDNA percentage. (DOCX)

S7 Table Results from comparisons between stable, HCV+, prior to rejection, and rejection episode samples. (DOCX)

S1 Text Study protocol (original; in German). (PDF)

S2 Text Study protocol (English translation). (PDF)

S3 Text Prospective analysis plan. (PDF)

## Abbreviations

ALT: alanine aminotransferase
AST: aspartate aminotransferase
ATG: antithymocyte globulin
AUC: area under the curve
BMI: body mass index
bpar: biopsy-proven acute rejection
cfDNA: cell-free DNA
ddPCR: droplet digital polymerase chain reaction
GcfDNA: graft-derived cell-free DNA
GLDH: glutamate dehydrogenase
HCV: hepatitis C virus
ISD: immunosuppressant drug
LFT: liver function test
LTx: liver transplantation
ROC: receiver operator characteristic
SNP: single nucleotide polymorphism
UKE: University Medical Center Hamburg-Eppendorf
UMG: University Medical Center Göttingen
WBC: white blood cell
γ-GT: γ-glutamyltransferase
