# Supplementary figures and images for: Graft-derived cell-free DNA, a noninvasive early rejection and graft damage marker in liver transplantation: A prospective, observational, multicenter cohort study
Source: PLoS Med. 2017 Apr 25;14(4):e1002286. doi: 10.1371/journal.pmed.1002286 (PMC5404754; doi:10.1371/journal.pmed.1002286)

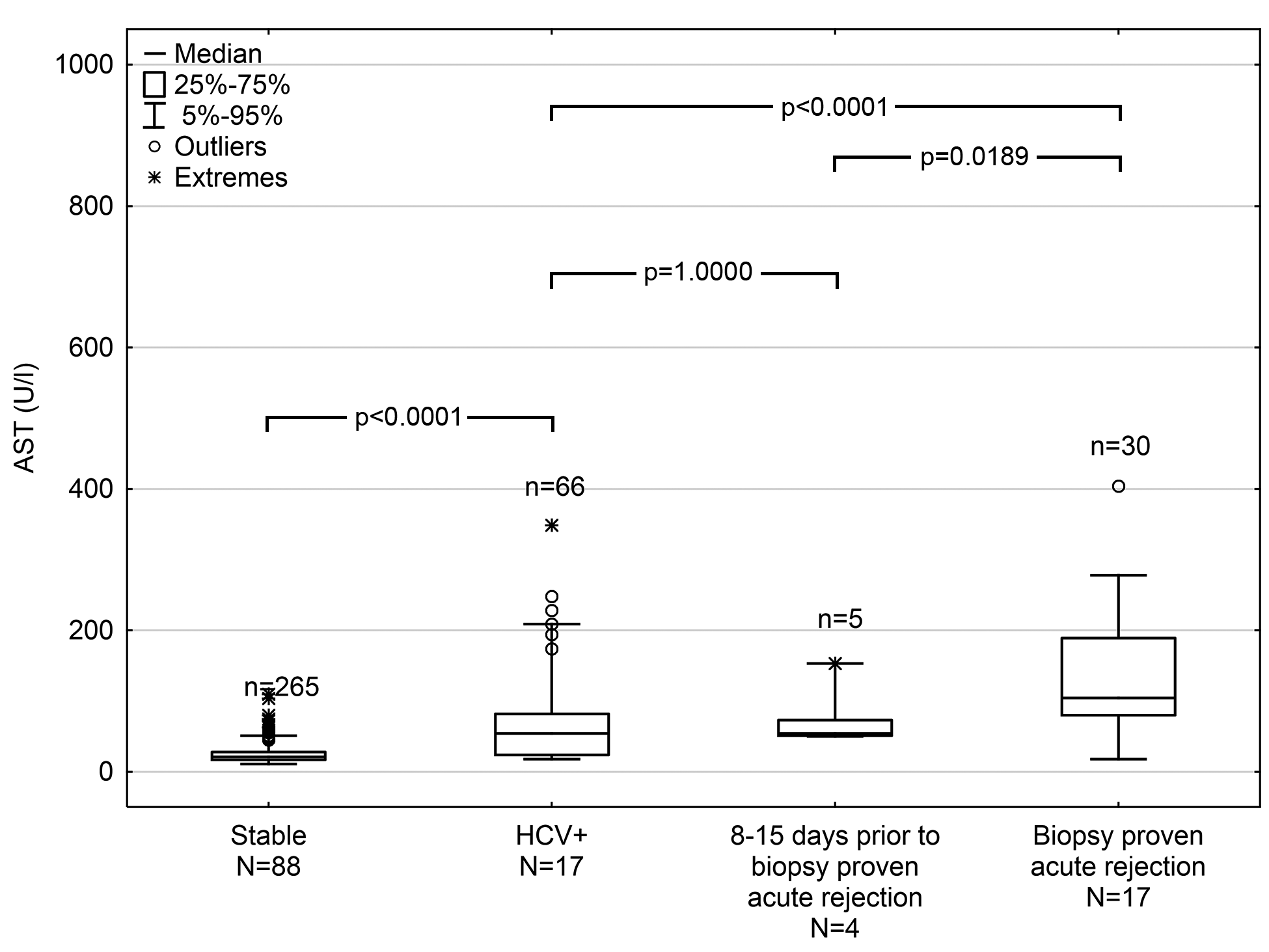

Supplement: S1 Fig — Boxes represent median with interquartile range, with whiskers showing the 5th–95th percentile and n’s showing the number of contributing values. N is the number of patients. (TIF) [file pmed.1002286.s002.tif]

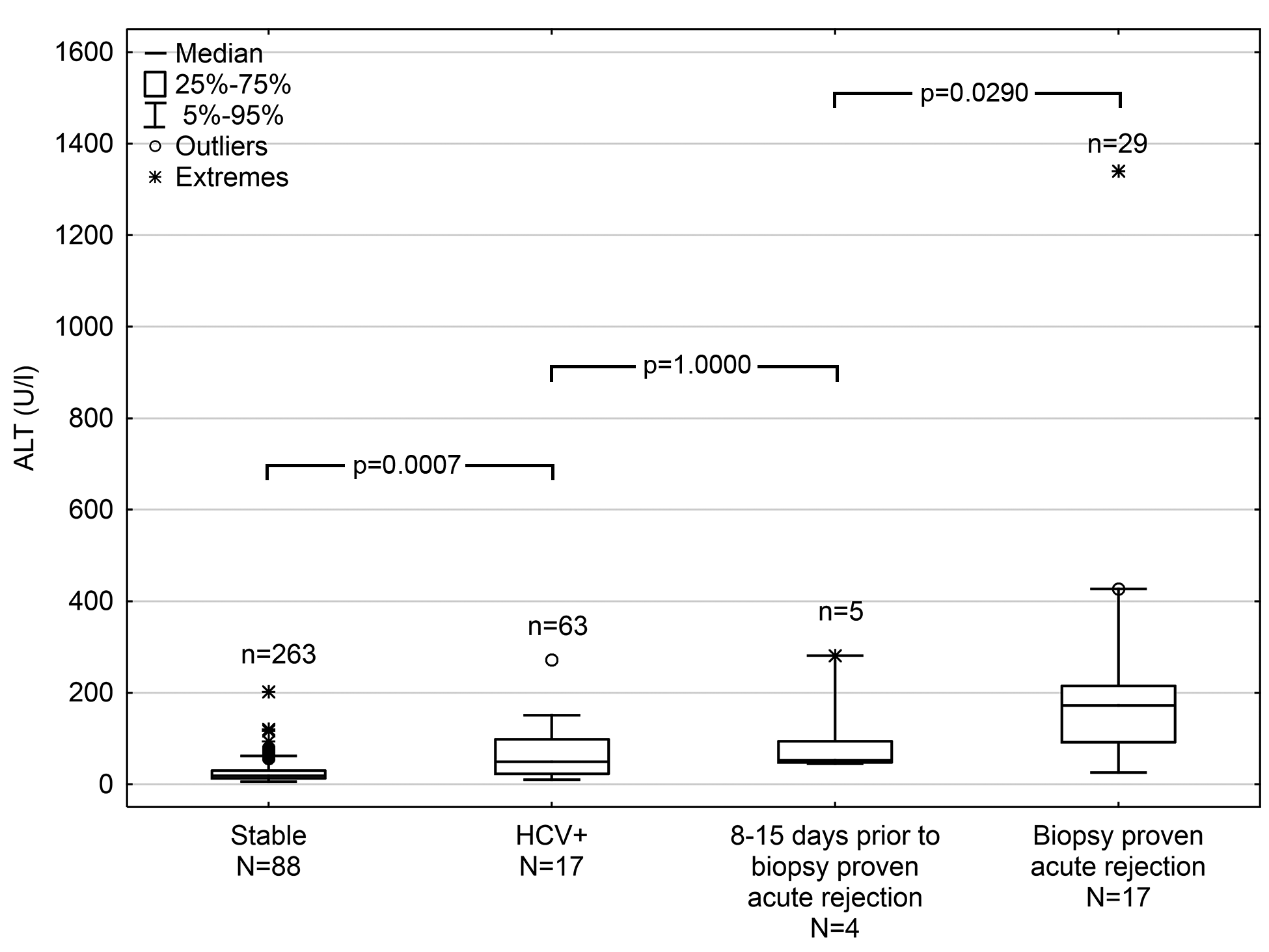

Supplement: S2 Fig — Boxes represent median with interquartile range, with whiskers showing the 5th–95th percentile and n’s showing the number of contributing values. N is the number of patients. (TIF) [file pmed.1002286.s003.tif]

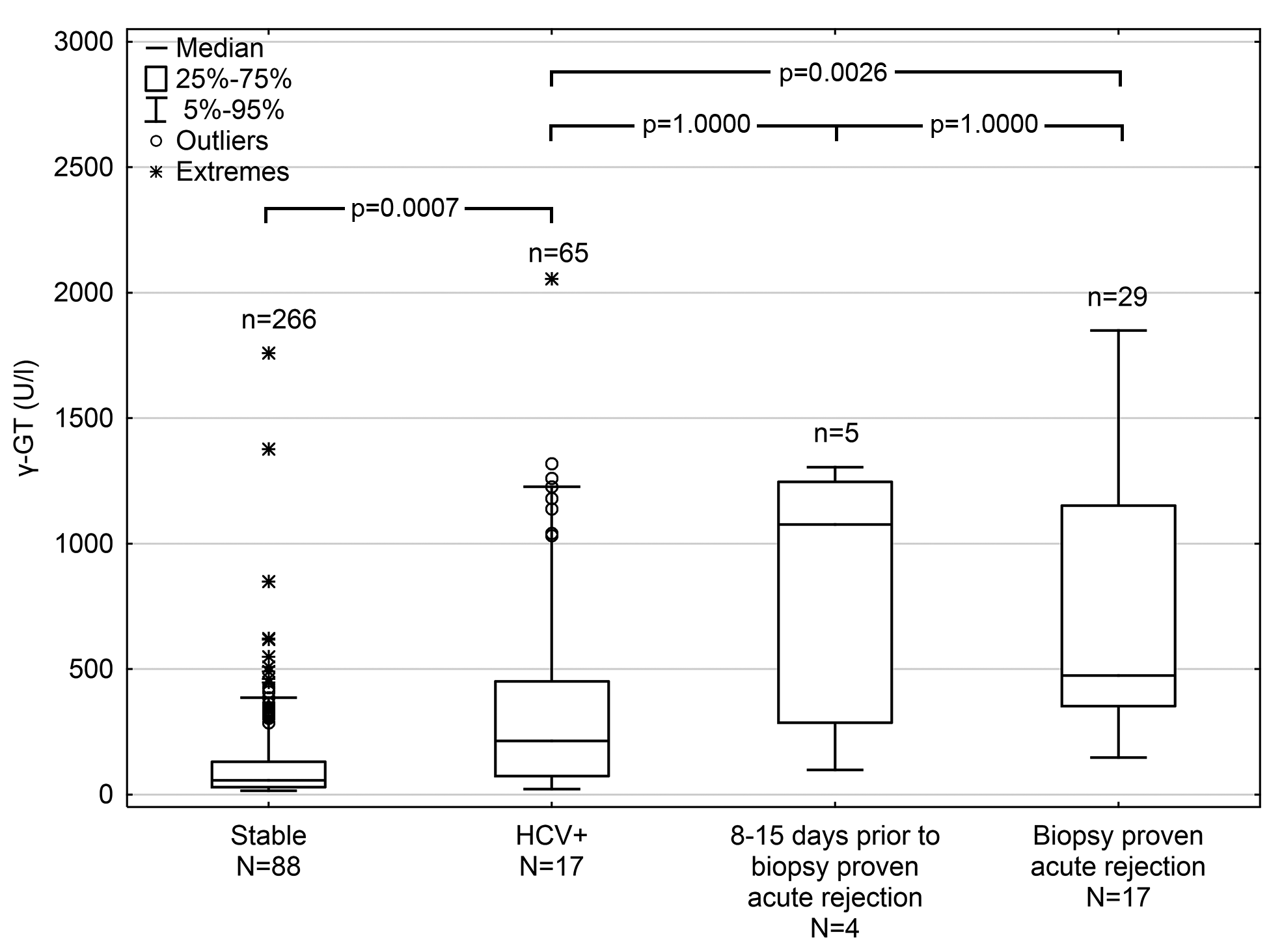

Supplement: S3 Fig — Boxes represent median with interquartile range, with whiskers showing the 5th–95th percentile and n’s showing the number of contributing values. N is the number of patients. (TIF) [file pmed.1002286.s004.tif]

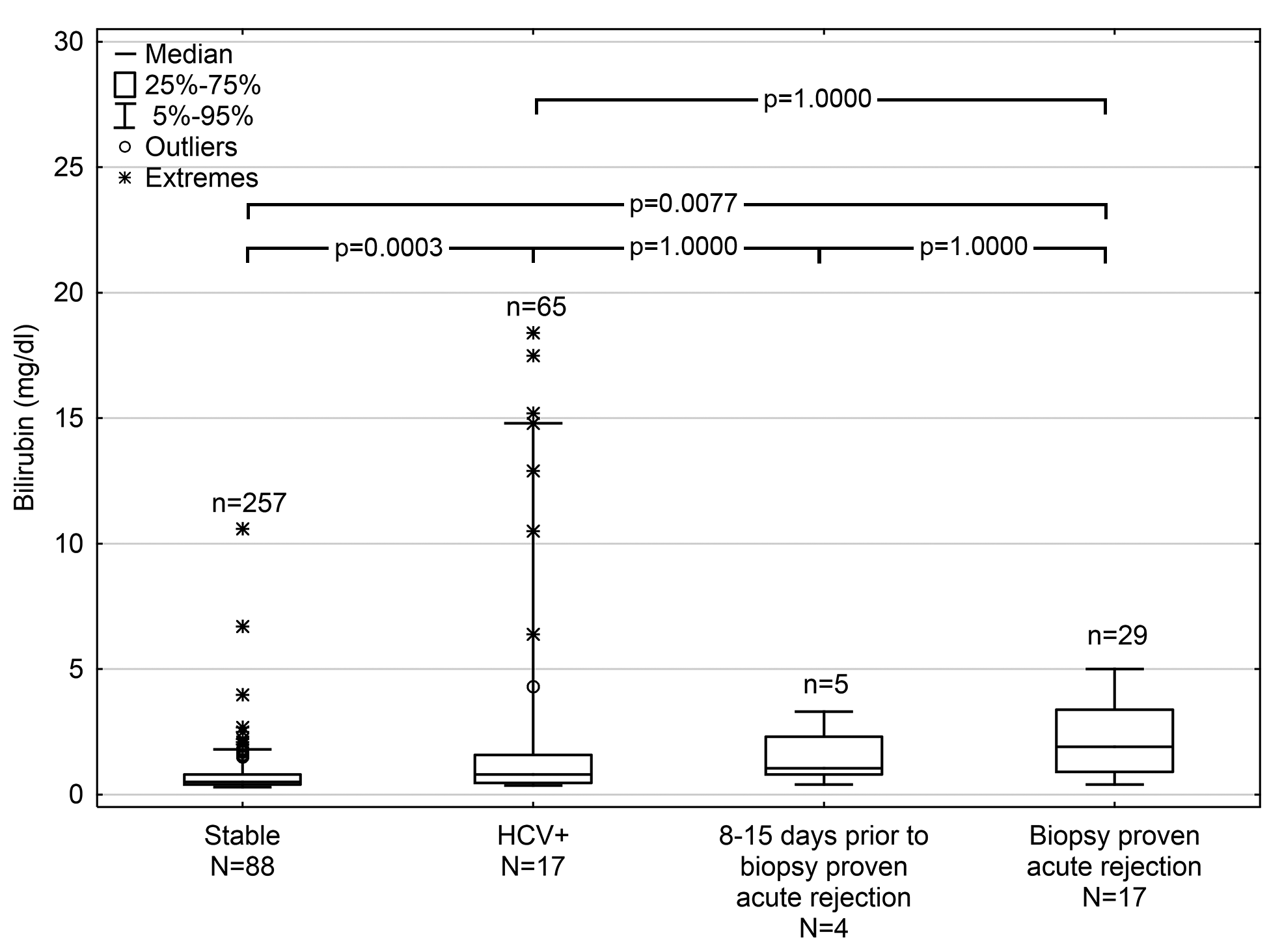

Supplement: S4 Fig — Boxes represent median with interquartile range, with whiskers showing the 5th–95th percentile and n’s showing the number of contributing values. N is the number of patients. (TIF) [file pmed.1002286.s005.tif]
