# Supplementary material for: Graft-derived cell-free DNA, a noninvasive early rejection and graft damage marker in liver transplantation: A prospective, observational, multicenter cohort study
Source: PLoS Med. 2017 Apr 25;14(4):e1002286. doi: 10.1371/journal.pmed.1002286 (PMC5404754; doi:10.1371/journal.pmed.1002286)
Supplement: S1 Table — (DOCX) [file pmed.1002286.s007.docx]

**Suppl. Table 1**

**Details for excluded patients**

| **N** | **Reason** |
| --- | --- |
| 4 | Case Report Form not properly filled out |
| 2 | Technical failure (clotted/DNA degraded) |
| 3 | Samples not properly labeled (WBC and plasma genotypes did not match) |
| 1 | WBC sample not received |
| 2 | Patients died immediately after surgery |
| 1 | Multi-transfusion syndrome (too much foreign DNA) |

WBC: white blood cell
