# Supplementary material for: Graft-derived cell-free DNA, a noninvasive early rejection and graft damage marker in liver transplantation: A prospective, observational, multicenter cohort study
Source: PLoS Med. 2017 Apr 25;14(4):e1002286. doi: 10.1371/journal.pmed.1002286 (PMC5404754; doi:10.1371/journal.pmed.1002286)
Supplement: S2 Table — (DOCX) [file pmed.1002286.s008.docx]

**Suppl. Table 2**

**Evaluated patients who died within the first year post LTx**

| **Patient** | **Center** | **Cause of death** | **POD** |
| --- | --- | --- | --- |
| 1 | UKE | Sepsis | 113 |
| 2 | UKE | HCC | 205 |
| 3 | UKE | Heart failure | 48 |
| 4 | UKE | Sepsis | 110 |
| 5 | UKE | Acute renal failure | 137 |
| 6 | UKE | *Unknown* | 116 |
| 7 | Charité | Sepsis | 49 |
| 8 | UMG | Reactive HCV infection | 345 |
| 9 | UMG | Infection | 57 |

POD: postoperative day, UKE: University Clinic Hamburg-Eppendorf, UMG: University Medical Center Goettingen
