# Supplementary material for: Graft-derived cell-free DNA, a noninvasive early rejection and graft damage marker in liver transplantation: A prospective, observational, multicenter cohort study
Source: PLoS Med. 2017 Apr 25;14(4):e1002286. doi: 10.1371/journal.pmed.1002286 (PMC5404754; doi:10.1371/journal.pmed.1002286)
Supplement: S3 Table — (DOCX) [file pmed.1002286.s009.docx]

**Suppl. Table 3**

**Patients with complicated course and who failed to meet pre-established criteria**

| **Patient** | **Number of visits** | **POD of testing** | **Major complications** | **GcfDNA (%)** |
| --- | --- | --- | --- | --- |
| 1 | 4 | 20; 48; 69; 181 | HCC (severely infiltrated); HBV+ | 16.9; 7.6; 13.9; 32.5 |
| 2 | 2 | 15; 43 | wound abscess | 38.3; 4.8 |
| 3 | 2 | 1; 22 | septicemia; pancreatitis | 79.0*; 5.6 |
| 4 | 1 | 9 | hepatic portal inflammation | 22.7 |
| 5 | 2 | 2; 15 | cholangitis | 82.7*; 10.4 |
| 6 | 4 | 9; 24; 59; 221 | rejection under steroids: day 9; ERCP: days 24 and 59; transplant failure due to cirrhosis: day 221 | 11.5; 14.4; 7.7; 33.9 |
| 7 | 3 | 2; 16; 43 | cholangitis | 88.1*; 7.9; 2.5 |
| 8 | 3 | 0; 6; 16 | rejection; cholangitis | 93.6; 82.1; 96.8 |

***** ischemia/reperfusion injury

POD: postoperative day, HCC: hepatocellular carcinoma; HBV, hepatitis B virus; ERCP: endoscopic retrograde cholangiopancreatography
