# Supplementary material for: Graft-derived cell-free DNA, a noninvasive early rejection and graft damage marker in liver transplantation: A prospective, observational, multicenter cohort study
Source: PLoS Med. 2017 Apr 25;14(4):e1002286. doi: 10.1371/journal.pmed.1002286 (PMC5404754; doi:10.1371/journal.pmed.1002286)
Supplement: S4 Table — (DOCX) [file pmed.1002286.s010.docx]

**Suppl. Table 4**

**Correlation between GcfDNA and conventional liver function tests**

**including GLDH in adult LTx patients from UKE/Hamburg-Eppendorf**

|  | **AST** | **ALT** | **γ-GT** | **GLDH** | **Bilirubin** |
| --- | --- | --- | --- | --- | --- |
| **GcfDNA**  p-value | 0.38 (0.22-0.52)  <.0001 | 0.63 (0.51-0.72)  <.0001 | 0.43 (0.28-0.56)  <.0001 | 0.49 (0.34-0.61)  <.0001 | 0.26 (0.08-0.41)  <.0001 |

Spearman correlation coefficients are given with CI^95%^; 125 samples

GcfDNA, Graft-derived cell-free DNA; AST, aspartate aminotransferase; ALT, alanine aminotransferase; γ-GT, γ-glutamyltransferase; GLDH, glutamate dehydrogenase
