# Supplementary material for: Graft-derived cell-free DNA, a noninvasive early rejection and graft damage marker in liver transplantation: A prospective, observational, multicenter cohort study
Source: PLoS Med. 2017 Apr 25;14(4):e1002286. doi: 10.1371/journal.pmed.1002286 (PMC5404754; doi:10.1371/journal.pmed.1002286)
Supplement: S5 Table — (DOCX) [file pmed.1002286.s011.docx]

**Suppl. Table 5**

**Diagnostic sensitivity at a 95% diagnostic specificity obtained from ROC curves in rejection vs. stable period samples after day 14 with data from UKE/Hamburg- Eppendorf**

|  | **n** | **AUC**  **%** | **CI^95%^**  **%** | **Sensitivity**  **(n=10)** | **CI^95%^**  **%** | **Specificity**  **(n=105)** | **CI^95%^**  **%** | **Threshold** |
| --- | --- | --- | --- | --- | --- | --- | --- | --- |
| **GcfDNA** | 115 | 98.3 | 96.2-100.0 | 90.0 | 55.5-99.7 | 96.2 | 90.5-99.0 | 12.4 (%) |
| **GLDH** | 115 | 98.2 | 96.2-100.0 | 80.0 | 44.4-97.5 | 96.2 | 90.5-99.0 | 20 (U/L) |

AUC, area under the curve; GcfDNA, Graft-derived cell-free DNA; GLDH, glutamate dehydrogenase
