# Supplementary material for: Graft-derived cell-free DNA, a noninvasive early rejection and graft damage marker in liver transplantation: A prospective, observational, multicenter cohort study
Source: PLoS Med. 2017 Apr 25;14(4):e1002286. doi: 10.1371/journal.pmed.1002286 (PMC5404754; doi:10.1371/journal.pmed.1002286)
Supplement: S6 Table — (DOCX) [file pmed.1002286.s012.docx]

**Suppl. Table 6**

**Additional multivariable logistic regression results of LFTs and GcfDNA**

| **Model** | **AIC** | **Parameter** | **Odds ratio** | **CI^95%^** | **p-value** |
| --- | --- | --- | --- | --- | --- |
| **LFTs with intercept including GcfDNA** | 50.7 | GcfDNA | 1.197 | 1.082-1.324 | 0.0005 |
|  |  | AST | 1.058 | 1.004-1.114 | 0.0356 |
|  |  | ALT | 0.993 | 0.966-1.021 | 0.6057 |
|  |  | γ-GT | 1.001 | 0.999-1.004 | 0.1782 |
|  |  | Bilirubin | 0.635 | 0.283-1.428 | 0.2724 |
| **LFTs with intercept without GcfDNA** | 65.2 | AST | 1.059 | 1.016-1.105 | 0.0074 |
|  |  | ALT | 1.009 | 0.986-1.032 | 0.4300 |
|  |  | γ-GT | 1.001 | 0.999-1.003 | 0.2265 |
|  |  | Bilirubin | 0.824 | 0.521-1.304 | 0.4082 |

GcfDNA, Graft-derived cell-free DNA; AST, aspartate aminotransferase; ALT, alanine aminotransferase; γ-GT, γ-glutamyltransferase; AIC, Akaike information criterion
