# Supplementary material for: Graft-derived cell-free DNA, a noninvasive early rejection and graft damage marker in liver transplantation: A prospective, observational, multicenter cohort study
Source: PLoS Med. 2017 Apr 25;14(4):e1002286. doi: 10.1371/journal.pmed.1002286 (PMC5404754; doi:10.1371/journal.pmed.1002286)
Supplement: S7 Table — (DOCX) [file pmed.1002286.s013.docx]

**Suppl. Table 7**

**Results from comparisons between stable, HCV positive, prior to rejection or rejection episode samples**

| **Laboratory Marker** | **Estimated difference** | **Adjusted CI^95%^*** | **p-value*** |
| --- | --- | --- | --- |
| **GcfDNA (%)** |  |  | <.0001 |
| Stable vs. HCV | 6.1 | 2.9-9.3 | <.0001 |
| Stable vs. prior to bpar | 30.8 | 21.7-39.9 | <.0001 |
| Stable vs. bpar | 28.3 | 24.3-32.2 | <.0001 |
| HCV vs. prior to bpar | 24.7 | 15.2-34.2 | <.0001 |
| HCV vs. bpar | 22.2 | 17.4-27.0 | <.0001 |
| Prior to bpar vs. bpar | -2.5 | -12.1-7.1 | 1.0000 |
| **AST (U/L)** |  |  | <.0001 |
| Stable vs. HCV | 43.5 | 25.2-61.9 | <.0001 |
| Stable vs. prior to bpar | 51.8 | 4.7-98.9 | 0.0225 |
| Stable vs. bpar | 106.5 | 85.5-127.5 | <.0001 |
| HCV vs. prior to bpar | 8.3 | -41.1-57.7 | 1.0000 |
| HCV vs. bpar | 62.9 | 36.9-88.9 | <.0001 |
| Prior to bpar vs. bpar | 54.6 | 5.9-103.4 | 0.0189 |
| **ALT (U/L)** |  |  | <.0001 |
| Stable vs. HCV | 41.6 | 13.2-69.9 | 0.0007 |
| Stable vs. prior to bpar | 79.0 | -9.0-167.1 | 0.1060 |
| Stable vs. bpar | 179.4 | 141-217.9 | <.0001 |
| HCV vs. prior to bpar | 37.5 | -53.3-128.3 | 1.0000 |
| HCV vs. bpar | 137.9 | 93.3-182.5 | <.0001 |
| Prior to bpar vs. bpar | 100.4 | 6.5-194.3 | 0.0290 |
| **γ-GT (U/L)** |  |  | <.0001 |
| Stable vs. HCV | 215.5 | 68.8-362.2 | 0.0007 |
| Stable vs. prior to bpar | 354.4 | 45.0-663.8 | 0.0154 |
| Stable vs. bpar | 470.7 | 328.6-612.7 | <.0001 |
| HCV vs. prior to bpar | 138.9 | -193.2-471.0 | 1.0000 |
| HCV vs. bpar | 255.1 | 64.8-445.5 | 0.0026 |
| Prior to bpar vs. bpar | 116.2 | -115.7-348.2 | 1.0000 |
| Bilirubin (U/L) |  |  | <.0001 |
| Stable vs. HCV | 1.39 | 0.49-2.29 | 0.0003 |
| Stable vs. prior to bpar | 0.62 | -1.75-2.98 | 1.0000 |
| Stable vs. bpar | 1.32 | 0.24-2.39 | 0.0077 |
| HCV vs. prior to bpar | -0.77 | -3.25-1.70 | 1.0000 |
| HCV vs. bpar | -0.07 | -1.38-1.23 | 1.0000 |
| Prior to bpar vs. bpar | 0.70 | -1.76-3.16 | 1.0000 |

***** p-value correction for pairwise comparisons based on Bonferroni procedure.

GcfDNA, Graft-derived cell-free DNA; bpar: biopsy-proven acute rejection; AST, aspartate aminotransferase; ALT, alanine aminotransferase; γ-GT, γ-glutamyltransferase
