# Supplementary material for: Graft-derived cell-free DNA, a noninvasive early rejection and graft damage marker in liver transplantation: A prospective, observational, multicenter cohort study
Source: PLoS Med. 2017 Apr 25;14(4):e1002286. doi: 10.1371/journal.pmed.1002286 (PMC5404754; doi:10.1371/journal.pmed.1002286)
Supplement: S1 Text — (PDF) [file pmed.1002286.s014.pdf]

Personalisierte Immunsuppression nach Lebertransplantation:

Eine Beobachtungsstudie zum Nutzen und zu therapeutischen Bereichen innovativer Biomarker

Prof. Dr. med. Otto Kollmar
Klinik für Allgemein- und Viszeralchirurgie
Universitätsmedizin Göttingen
Robert-Koch-Str. 40
Tel: +49 551 39 2260
Fax: +49 551 39 2202
otto.kollmar@med.uni-goettingen.de

Dr. med. Jochen Gaedcke
Klinik für Allgemein- und Viszeralchirurgie
Universitätsmedizin Göttingen
Robert-Koch-Str. 40
Tel: +49 551 39 6944
Fax: +49 551 39 12550
jochen.gaedcke@med.uni-goettingen.de

<u>Laboranalysen:</u>
Herr Prof. Dr. Dr. med. Michael Oellerich
Abteilung für Klinische Chemie, Universitätsmedizin Göttingen, Robert-Koch-Str. 40, 37075 Göttingen
Tel: +49 551 39 8561
Fax: +49 551 39 8551 (zentrales Studienfaxgerät: 0551 39 22194)
michael.oellerich@med.uni-goettingen.de

<u>Studienkoordination und Monitoring:</u>
Prof. Dr. med. Jürgen Brockmöller
Abteilung Klinische Pharmakologie, Universitätsmedizin Göttingen, Robert-Koch-Str. 40, 37075 Göttingen
Tel: +49 551 39 5770
Fax: +49 551 39 12767
jbrockm@gwdg.de

Vertrauliche Informationen: Weitergabe außerhalb der Studiendurchführung ohne Genehmigung der Autoren verboten.

Inhalt

1. Allgemeine Informationen ... 5
1.1. Studientitel und Protokollversionen ... 5
1.2. Sponsor und Monitoring ... 5
1.3. Verantwortliche Ärzte/innen und Wissenschaftler/Innen ... 5
1.4. Medizinisch-wissenschaftlicher Beirat ... 7
2. Einführung: Medizinischer und wissenschaftlicher Hintergrund ... 7
2.1. Immunsuppression nach Lebertransplantation ... 7
2.2. Gegenwärtiger Stand des therapeutischen Arzneimittelkonzentrations-Monitoring (TDM) nach Lebertransplantation ... 8
2.3. Gegenwärtiger Stand des therapeutischen Immunmonitoring nach Lebertransplantation ... 8
2.4. Gegenwärtiger Stand pharmakogenetischer Parameter in der Immunsuppression ... 10
2.5. Weitere Informationen zur hier untersuchten Patientengruppe ... 10
3. Ziele der Studie ... 10
4. Studiendesign ... 12
4.1. Primäre und sekundäre Endpunkte ... 12
4.2. Studiendesign ... 13
4.3. Maßnahmen gegen systematische Fehler bzw. Verzerrungen in den Ergebnissen 13
4.4. Untersuchte Biomarker ... 14
4.5. Studiendauer und Zeitplan ... 15
4.6. Abbruchkriterien ... 16
4.7. Datenmanagement ... 17
5. Auswahl und Ausschluß von Studienteilnehmern ... 17
5.1. Einschlußkriterien ... 17
5.2. Ausschlußkriterien ... 17
5.3. Kriterien für individuellen Studienabbruch und Dokumentation in diesen Fällen ... 17
6. Arzneitherapie und weitere Behandlung ... 17
7. Statistik ... 18
7.1. Statistische Datenauswertung ... 18
7.2. Begründung für Fallzahl ... 18
7.3. Kriterien für Studienabbruch ... 18
7.4. Umgang mit fehlenden und unvollständigen Daten ... 18
7.5. Änderungen am statistischen Auswertekonzept ... 19
7.6. Kriterien für Ausschluss von Daten und Patienten von der statistischen Auswertung und Publikation ... 19
8. Zugang zu Quelldaten ... 19
9. Maßnahmen zur Sicherung der Datenqualität ... 19
10. Ethische und rechtliche Gesichtspunkte ... 20
11. Datenmanagement ... 20
12. Finanzierung der Studie und Versicherung ... 20
13. Publikationsvereinbarungen ... 20
14. Zitierte Literatur ... 21

Vertrauliche Informationen: Weitergabe außerhalb der Studiendurchführung ohne Genehmigung der Autoren verboten.

Synopsis

**Titel**	**Personalisierte Immunsuppression nach Lebertransplantation: Eine Beobachtungsstudie zum Nutzen und zu therapeutischen Bereichen innovativer Biomarker**<br><br>**(**Biomarker assisted personalized immunosuppression following liver transplantation)
**Studiendesign**	Prospektive nicht-interventionelle Beobachtungsstudie (Kohortenstudie)
**Hintergrund**	Eine immunsuppressive Therapie nach Transplantation stellt eine Gratwanderung zwischen Infektion (Überdosierung) auf der einen und Rejektion (Unterdosierung) auf der anderen Seite dar.<br><br>Die Aufnahme von vielen Immunsuppressiva in die relevanten Immunzellen und die Ausscheidung aus den Zellen ist sehr variabel. Daher erlaubt die Messung intrazellulärer Zielkonzentrationen der Immunsuppressiva in Zukunft wahrscheinlich eine deutlich bessere individuelle Therapieanpassung als gegenwärtig praktizierte Formen des Therapeutischen Drug Monitoring.<br><br>Wir gehen davon aus, dass es auch bei gleichen Blut- und Gewebsspiegeln individuell erhebliche Unterschiede in der Immunsuppression gibt. Daher erlaubt die Messung weiterer Immun-Biomarker in Zukunft wahrscheinlich eine bessere individuelle Therapieanpassung.
**Studien-finanzierung**	Die Studie wird durch das Bundesministerium für Bildung und Forschung gefördert (Förderkennzeichen 01ES1102).
**Studienziele**	Im Rahmen der medizinisch indizierten Therapie sollen Datenanalysen und ergänzende Laboranalysen an einer relativ großen Zahl von Patienten nach Lebertransplantation zu folgenden Zielen beitragen.<br><br>• Korrelation der intrazellulären Konzentrationen in den mononukleären Blutzellen (itc-TDM) mit den Konzentrationen im nicht fraktionierten Blut (TDM) sowie Definition therapeutische Zielbereiche intrazellulärer Konzentrationen von Immunsuppressiva<br><br>• Exploration von innovativen Biomarkern für Immunzell-Aktivierungs-Monitoring (insbesondere IL2-CD8 und CD4-Aktivierung) und Immuntoleranz-Monitoring (Insbesondere FOXP3 und B-Zell-Differenzierungsgene) sowie weiterer im Blutplasma nachweisbarer Zytokine.<br><br>• Messung von im Plasma zirkulierender DNA (CNA) differenziert nach DNA von Organspender und Empfänger bezüglich des möglichen diagnostischen Wertes in der Früherkennung und Differentialdiagnose von Rejektionen.<br><br>• Exploration des möglichen diagnostischen Wertes pharmakogenomischer Biomarker für die Individualisierung der immunsuppressiven Therapie.<br><br>Die Referenzbereiche, die in vorliegender Studie identifiziert werden und die Marker, die sich als informativ herausstellen, sollen nach Bestätigung in einer Nachfolgestudie dann dazu beitragen, dass die Immunsuppression von Patienten nach Lebertransplantation effektiver und vor allem nebenwirkungsärmer und damit sicherer wird.
**Endpunkte**	• Primärer Endpunkt: Messung der intrazellulären Konzentrationen in den mononukleären Blutzellen (itc-TDM) und deren Korrelation zu den Konzentrationen im nicht fraktionierten Blut (TDM)<br>• Sekundäre Endpunkte: Infektionen und Rejektionen nach Lebertransplantation sowie klinischen und labormedizinische Parameter, die Hinweise auf Unter- oder Überimmunsuppression geben und Nebenwirkungen der Medikamente
**Patientenzahl**	• Patientenzahl: 125 Patienten nach Lebertransplantation

Vertrauliche Informationen: Weitergabe außerhalb der Studiendurchführung ohne Genehmigung der Autoren verboten.

**und Studiendauer**	• Studienorte: Universitätsmedizin Göttingen, Klinik für Allgemein- und Viszeralchirurgie, Universitätsklinikum Hamburg Eppendorf, Klinik und Poliklinik für Hepatobiliäre Chirurgie und Transplantationschirurgie und Charité Berlin, Campus Virchow-Klinikum, Klinik für Allgemein-, Visceral- und Transplantationschirurgie<br>• Studiendauer: 18 Monate bei einer Beobachtungsdauer je Patient von 12 Monaten
**Einschluss-kriterien**	• Einwilligungsfähige Patienten innerhalb der ersten 9 Monate nach Lebertransplantation<br>• Behandlung mit wenigstens einem der Arzneimittel Cyclosporin A, Tacrolimus, Mycophenolsäure, Everolimus (letzteres erst und nur sofern eine Zulassung für die Therapie nach Lebertransplantation in Deutschland besteht)
**Ausschluss-kriterien**	• Teilnahme an Therapiestudien zu anderen immunsuppressiven Medikamenten (Ausnahme: Sponsor stimmt der Teilnahme zu)<br>• Fehlende Einwilligungsfähigkeit<br>• Alter < 18 Jahre
**Statistische Daten-auswertung**	Prof. Dr. Tim Friede<br>Abteilung Medizinische Statistik<br>Universitätsmedizin Göttingen<br>Humboldallee 32<br>37075 Göttingen<br>Tel: +49 551 39 4990<br>Fax: +49 551 39 4995<br>tim.friede@med.uni-goettingen.de
**Leitung**	Prof. Dr. med. Otto Kollmar<br>Klinik für Allgemein- und Viszeralchirurgie<br>Universitätsmedizin Göttingen<br>Robert-Koch-Str. 40<br>Tel: +49 551 39 2260<br>Fax: +49 551 39 2202<br>otto.kollmar@med.uni-goettingen.de<br><br>Dr. med. Jochen Gaedcke<br>Klinik für Allgemein- und Viszeralchirurgie<br>Universitätsmedizin Göttingen<br>Robert-Koch-Str. 40<br>Tel: +49 551 39 6944<br>Fax: +49 551 39 12550<br>jochen.gaedcke@med.uni-goettingen.de<br><br>Laboranalysen:<br>Herr Prof. Dr. Dr. med. Michael Oellerich<br>Abteilung für Klinische Chemie<br>Universitätsmedizin Göttingen<br>Robert-Koch-Str. 40<br>37075 Göttingen<br>Tel: +49 551 39 8561<br>Fax: +49 551 39 8551<br>michael.oellerich@med.uni-goettingen.de<br><br>Verantwortlich für Studienkoordination und Monitoring:<br>Prof. Dr. med. Jürgen Brockmöller<br>Abteilung Klinische Pharmakologie<br>Universitätsmedizin Göttingen<br>Robert-Koch-Str. 40<br>37075 Göttingen<br>Tel: +49 551 39 5770<br>Fax: +49 551 39 12767<br>jbrockm@gwdg.de

Vertrauliche Informationen: Weitergabe außerhalb der Studiendurchführung ohne Genehmigung der Autoren verboten.

1. Allgemeine Informationen

1.1. Studientitel und Protokollversionen

Personalisierte Immunsuppression nach Lebertransplantation:
Eine Beobachtungsstudie zum Nutzen
und zu therapeutischen Bereichen innovativer Biomarker

Acronym: PI-LTX

Protokollversion: 1.0

1.2. Sponsor und Monitoring

Es handelt sich um eine multizentrische akademisch initiierte und nicht interventionelle Beobachtungsstudie zum Immunsuppressions-Monitoring nach Lebertransplantation.

Die Durchführung der Studie ist sichergestellt durch die Förderung seitens des Bundesministerium für Bildung und Forschung (Förderkennzeichen 01ES1102) und durch ein darüber hinausgehendes Engagement die beteiligten medizinisch-wissenschaftlichen Einrichtungen.

Das Monitoring wird durch eine/n entsprechend ausgebildete/n Wissenschaftler/in aus der Abteilung klinische Pharmakologie der Universitätsmedizin Göttingen durchgeführt.

1.3. Verantwortliche Ärzte/innen und Wissenschaftler/Innen

Prof. Dr. med. Otto Kollmar
Klinik für Allgemein- und Viszeralchirurgie
Universitätsmedizin Göttingen
Robert-Koch-Str. 40
Tel: +49 551 39 2260
Fax: +49 551 39 2202
otto.kollmar@med.uni-goettingen.de

Dr. med. Jochen Gaedcke
Klinik für Allgemein- und Viszeralchirurgie
Universitätsmedizin Göttingen
Robert-Koch-Str. 40
Tel: +49 551 39 6944
Fax: +49 551 39 12550
jochen.gaedcke@med.uni-goettingen.de

Zusätzlich zu den hier genannten Personen werden an der Beobachtungsstudie weitere Personen mitarbeiten. Diese werden am jeweiligen Zentrum in einer Delegationsliste eingetragen und die jeweiligen Recht, Aufgaben und Pflichten werden schriftlich festgehalten.

Vertrauliche Informationen: Weitergabe außerhalb der Studiendurchführung ohne Genehmigung der Autoren verboten.

Verantwortlich für multizentrische Studienkoordination und Monitoring:

Herr Prof. Dr. med. Jürgen Brockmöller
Abteilung Klinische Pharmakologie
Universitätsmedizin Göttingen
Robert-Koch-Str. 40
37075 Göttingen
Tel: +49 551 39 5770
Fax: +49 551 39 12767
jbrockm@gwdg.de

Laboranalysen
Wissenschaftliche Koordination:
Herr Prof. Dr. Dr. med. Michael Oellerich
Abteilung für Klinische Chemie
Universitätsmedizin Göttingen
Robert-Koch-Str. 40
37075 Göttingen
Tel: +49 551 39 8561
Fax: +49 551 39 8551
michael.oellerich@med.uni-goettingen.de

Innerhalb der Abteilung Klinische Chemie: Molekular-Immunologische Analytik:
Herr PD Dr. med. Gunnar Brandhorst
Abteilung für Klinische Chemie
Universitätsmedizin Göttingen
Robert-Koch-Str. 40
37075 Göttingen
Tel: +49 551 39 8062
Fax: +49 551 39 8551
gunnar.brandhorst@med.uni-goettingen.de

Innerhalb der Abteilung Klinische Chemie: Arzneimittel-Konzentrationsanalytik:
Herr Dr. rer. nat. Frank Streit
Abteilung für Klinische Chemie
Universitätsmedizin Göttingen
Robert-Koch-Str. 40
37075 Göttingen
Tel: +49 551 39 5792
Fax: +49 551 39 8551
fstreit@med.uni-goettingen.de

Statistische Planung und Auswertung:
Herr Prof. Dr. Tim Friede
Abteilung Medizinische Statistik
Universitätsmedizin Göttingen
Humboldtallee 32
37073 Göttingen
Tel: +49 551 39 4990
Fax: +49 551 39 4995
tim.friede@med.uni-goettingen.de

Vertrauliche Informationen: Weitergabe außerhalb der Studiendurchführung ohne Genehmigung der Autoren verboten.

1.4. Medizinisch-wissenschaftlicher Beirat

Der Studienverlauf und alle Protokolländerungen werden mit dem medizinisch-wissenschaftlichen Beirat abgestimmt. Der medizinisch-wissenschaftliche Beirat umfasst die 1.1. bis 1.4 genannten Personen. Weitere Personen können nach einstimmiger Absprache in diesen Beirat aufgenommen werden.

Ein *independent data safety monitoring board* ist nicht erforderlich bei der Beobachtungsstudie, die nur mit minimalen Risiken und Belastungen einhergeht.

2. Einführung: Medizinischer und wissenschaftlicher Hintergrund

2.1. Immunsuppression nach Lebertransplantation

In der Regel benötigen Patienten nach Lebertransplantation eine langfristig täglich einzunehmende Basis-Immunsuppression. Im Rahmen der Basis-Immunsuppression werden je nach individueller Konstellation typischerweise Kombinationen aus zwei oder drei der folgenden Medikamente eingesetzt: a) Einem so genannten Calcineurininhibitor (Cyclosporin oder Tacrolimus), b) einem Glukokortikoid (Prednison oder Methylprednisolon), und c) einem Antimetaboliten (Mycophenolatmofetil oder Azathioprin). Gegenstand vorliegender Beobachtungsstudie sind ausschließlich Therapien mit Immunsuppressiva, die im Rahmen der Lebertransplantation zugelassen sind.

Unmittelbar vor bzw. in den Wochen nach Transplantation sowie bei akuten Organabstoßungen (Rejektionen) kommen weitere sehr ausgeprägt immunsuppressiv wirkende Medikamente als Induktions- und Rejektionstherapie zum Einsatz.

Mit der Basis-Immunsuppression lässt sich eine Organabstoßung in der Regel verhindern. Dennoch ist diese immunsuppressive Therapie eine enge Gratwanderung zwischen Unterdosierung mit allen Risiken der Organabstoßung auf der einen Seite und einer Überdosierung mit den Risiken von möglicherweise lebensbedrohlichen Infektionen und dosisabhängigen Arzneimittelnebenwirkungen.

Für die Auswahl und Dosierung der individuell optimalen Immunsuppression existieren umfangreiche klinische Erfahrungen. Dennoch ist das individuelle Ansprechen auf die immunsuppressiven Medikamente nur begrenzt vorhersagbar und auch beim gleichen Patienten kann der Bedarf an Immunsuppressiva im Laufe der Monate und Jahre nach Transplantation erheblich schwanken. Verlässliche, allgemein in der medizinischen Praxis akzeptierte Indikatoren, ob ein Patient zu einem bestimmten Zeitpunkt richtig immunsupprimiert ist, also weder unter- noch übertherapiert ist, existieren gegenwärtig nicht.

Verlässlich sind die so genannten *klinischen Endpunkte* Rejektion und Infektion sowie Nebenwirkungen der Medikamente. Indes möchte man es nicht zu diesen so genannten klinischen Endpunkten kommen lassen, da diese sich unter Umständen nur schwer beherrschen lassen oder durch Notwendigkeit intensiver Behandlungen und Hospitalisierungen die Lebensqualität der Patienten beeinträchtigten. Also bedient man sich so genannter Surrogatparameter, also Hilfsgrößen, die wichtige Teilhinweise geben können, ob ein Patienten die richtige Dosis bzw. die richtige Auswahl der Medikamente bekommt. Seit langem etabliert als Surrogatparameter sind labormedizinische Marker für Organfunktionsstörungen und das therapeutische Drug Monitoring (Arzneimittel-Konzentrations-Monitoring).

Vertrauliche Informationen: Weitergabe außerhalb der Studiendurchführung ohne Genehmigung der Autoren verboten.

2.2. Gegenwärtiger Stand des therapeutischen Arzneimittelkonzentrations-Monitoring (TDM) nach Lebertransplantation

Die individuelle Einstellung der Dosis von Medikamenten wie Cyclosporin, Tacrolimus oder Everolimus orientiert an den Blut- oder Plasmakonzentrationen (*therapeutic drug monitoring* bzw. *Blutspiegelmessungen*) und ist therapeutischer Standard in der Transplantationsmedizin (Holt *et al.* 2002) und wird auch in entsprechenden Fachinformationen z.B. von Tacrolimus empfohlen. Diese Einschätzung ergibt sich unter anderem daraus, dass die Blutspiegel besser als die Dosis mit Wirkungen und Nebenwirkungen korrelieren als die Dosis allein (Shaw *et al.* 1998) und gut individuelle Überdosierungen erkennen lassen. Individuelle Überdosierungen können akut z.B. in Form von Infektionen und langfristig z.B. in Form von Krebserkrankungen fatale Konsequenzen haben. Insofern wird das therapeutische Drug Monitoring in diesem Bereich als obligatorisch angesehen (Touw *et al.* 2005).

Nichtsdestotrotz gibt es weiterhin insbesondere in den ersten Monaten nach Transplantation viele Komplikationen auf Grund einer individuellen Unter- oder Über-Immunsuppression.

Ein seit langem bekannter Schwachpunkt des gegenwärtig praktizierten TDM ergibt sich daraus, dass die Medikamente nicht dort analysiert werden, wo sie eigentlich wirken, nämlich innerhalb der Immunzellen, sondern aus praktischen Gründen entweder im Blutplasma (also außerhalb der Zellen) oder im Gesamtblut. Da aber die Aufnahme von Medikamenten in die Zellen und die Ausscheidung aus Zellen aus sehr unterschiedlichen Gründen (zum Beispiel genetische Variation und Wechselwirkungen mit anderen Medikamenten und Nahrungsstoffen) sehr variabel ist, gibt es erste Hinweise aus klinischen Studien (z.B. (Crettol *et al.* 2008; Falck *et al.* 2008)) und viele weitere Gründe für unsere Vermutung, dass die Messung intrazellulärer Zielkonzentrationen der Immunsuppressiva (itc-TDM, *intracellular target concentration therapeutic drug monitoring*) eine deutlich bessere individuelle Therapieanpassung erlaubt als die gegenwärtige Praxis der Messung von Gesamtblut- oder Plasmakonzentrationen.

Unsere Forschung soll langfristig zur Klärung beitragen, ob diese Messung der Immunsuppressiva an den Zielzellen tatsächlich zur einer Verbesserung der Therapie führen kann. Das hier durchgeführte Projekt soll zunächst die wichtigen Informationen liefern, welche intrazellulären Konzentrationen in der Regel mit guter Wirkung und wenig Nebenwirkungen einhergehen.

2.3. Gegenwärtiger Stand des therapeutischen Immunmonitoring nach Lebertransplantation

Gut kontrollierte Medikament- bzw. Wirkstoffkonzentrationen am Zielort sind nur eine von vielen Voraussetzungen für gute therapeutische Wirkungen. Daher wird versucht, durch Laborparameter die Aktivität des Immunsystems zu erfassen. Hier lassen sich drei Arten von teilweise überlappenden Parametern bzw. Zielen unterschieden:

a) Immunzell-Aktivitätsmarker (Rejektions- oder Infektions-Früherkennungsmarker)
b) Immuntoleranzmarker
c) Weitere Marker zur Früherkennung und Differentialdiagnose von Transplantat-Funktionsstörungen

Vertrauliche Informationen: Weitergabe außerhalb der Studiendurchführung ohne Genehmigung der Autoren verboten.

Dabei geht es also darum, mit a) Markern der Immunzellaktivität (Rejektions/Infektions-Früherkennungsmarkern) bereits möglichst lange vor einer offenkundig erkennbaren Störung die Zeichen einer Unter- oder Über-Immunsuppression zu erkennen, um so das Auftreten von schweren Infektionen und Rejektionen zu verhindern. Ferner geht es darum, mit b) Immuntoleranz-Markern Patienten zu identifizieren, die eine gute Toleranz für das transplantierte Organ haben und insofern von einer niedrigen Dosis der Immunsuppressiva profitieren würden. Und bei den c) Markern zur Differentialdiagnose von Transplantatfunktions-Störungen geht es darum, zwischen einer Arzneimitteltoxizität bzw. Überdosierung und einer Rejektion zu differenzieren, ohne dass belastende Organbiopsien erforderlich sind. Insbesondere folgende Parameter sollen hier gemessen werden:

Marker der Immunzellaktivität (Rejektions/Infektions-Früherkennungsmarker)

Cylex® ImmuKnow® - $CD4^+$ Immunantwort. Die Bestimmung der ATP-Synthese in $CD4^+$ T-Zellen nach Stimulation mit Phythämagglutinin dient der Abschätzung der globalen T-Zell-Immunantwort. Das Verfahren scheint bei Patienten nach Organtransplantation für die Vorhersage von Infektionen und ggf. auch Abstoßungsreaktionen geeignet zu sein (Kowalski *et al.* 2003; Schulz-Juergensen *et al.* 2012).

IL-2/$CD8^+$ In einzelnen Studien wurde die Interleukin-2 (IL-2) Sekretion in $CD8^+$ T-Zellen nach Stimulation *ex vivo* als prädiktiver Marker für die Transplantabstossung identifiziert (Boleslawski *et al.* 2004; Millan *et al.* 2010). Hierbei wird durchflußzytometrisch die Anzahl IL-2-exprimierender Zellen nach Sekretionshemmung im Verhältnis zur Gesamtzahl der $CD8^+$ T-Zellen ermittelt.

Immuntoleranzmarker

B-Zell-Differenzierungsgene. Die Untersuchung der Genexpression von IGKV1D-13, IGKV4-1 und IGLL1 in B-Zellen ist ein potentielles Verfahren für die Identifikation operational toleranter Patienten und kann mittels quantitativer PCR durchgeführt werden. In einem Kollektiv von Patienten nach Nierentransplantation konnte anhand der Expression dieser Gene zwischen toleranten und nicht-toleranten Patienten unterschieden werden (Newell *et al.* 2010).

FoxP3-Expression zur Quantifizierung regulatorischer T-Zellen (Tregs). Das Forkhead Box P3 (Foxp3) wird in regulatorischen T-Zellen (Tregs) exprimiert, deren Anzahl mit dem Vorkommen einer operationalen Toleranz (d.h. mehr als 1 Jahr keine Abstoßung ohne immunsuppressive Medikation) bei Patienten nach Organtransplantation zu korrelieren scheint. Derzeit wird vor allem die Bestimmung $CD4^+CD25^{high}Foxp3^+$ Tregs in mononukleären Zellen mittels intrazellulärer Fluoreszenzfärbung (FACS) zur Quantifizierung der Tregs herangezogen (Pons *et al.* 2008). Da auch aktivierte T-Zellen transient FoxP3 exprimieren können, wurde zur Verbesserung der analytischen Spezifität vorgeschlagen, die Quantifizierung einer FoxP3-spezifischen Demethylierung als Surrogatparameter für die Bestimmung der Tregs zu verwenden (Wieczorek *et al.* 2009).

Marker zur Differentialdiagnose von Transplantatfunktions-Störungen

Donor-spezifische zirkulierende DNA (CNA). Auf Grund genetischer Unterschiede zwischen Transplantatspender und -empfänger kann das Verhältnis von zirkulierender Spender- und Empfänger-DNA im zellfreien Plasma der Patienten möglicherweise sowohl die Schwere als auch den Verlauf einer Abstoßungsreaktion beschreiben. Bei einer Rejektion kommt es durch die Immunreaktion zu einer Apoptose von Spenderorganzellen

Vertrauliche Informationen: Weitergabe außerhalb der Studiendurchführung ohne Genehmigung der Autoren verboten.

und die dabei freigesetzte DNA wird im Plasma quantifiziert. Die Sequenzierung der zirkulierenden Plasma-DNA (CNA) mit anschließender Quantifizierung stellt eine potentielle Alternative zu aufwändigen und invasiven Biopsien dar (Snyder *et al.* 2011).

Bei letzteren Analysen handelt es sich in der Zielrichtung nicht um genetische Analysen von Markern, die eine spezifische Bedeutung für Phänotypen haben, , dennoch kann die Erkennbarkeit von Erbinformation bei diesen Analysen nicht verhindert werden, so dass wir diese Messungen in der Patienteninformation unter den pharmakogenetischen Analysen nennen.

2.4. Gegenwärtiger Stand pharmakogenetischer Parameter in der Immunsuppression

Für eine Reihe von Immunsuppressiva gehört die pharmakogenetische Typisierung zunehmend zum medizinischen Alltag. Das gilt insbesondere für die Testung hinsichtlich der Thiopurin-S-Methyltransferaseaktivität bei Azathioprin (Schütz *et al.* 1995; Compagni *et al.* 2008), die Testung der CYP3A5-Aktivität bei Tacrolimus (Kamdem *et al.* 2005) und möglicherweise auch Varianten im Enzym CYP3A4 (Varianten **1B*, *22) für Cyclosporin und Tacrolimus. Andere Testungen z.B. auf Varianten des Arzneimitteltransporters MDR1 erscheinen nach einigen Studien sehr interessant, sollten aber weiterhin eher im Rahmen von Forschungsprojekten erfolgen, da die Ergebnisse kontrovers sind. Wir möchten hier mittels Einzelnukleotidpolymorphismus-Analysen (SNP) und Resequenzierung ein sehr breites Spektrum möglicher Kandidatengen-Varianten bezogen auf die Fragestellungen der hier vorliegenden Studie umfassend analysieren.

2.5. Weitere Informationen zur hier untersuchten Patientengruppe

Die Patienten sollen vorzugsweise möglichst früh in den ersten 2 Wochen nach Lebertransplantation in die Studie eingeschlossen werden (sobald nach der Operation ein entsprechend stabiler Gesundheitszustand erreicht ist und die Patienten einwilligungsfähig sind). Ein Einschluss kommt bis zum Ende des 9. Monats nach Transplantation in Frage, die Beobachtungszeit pro Patient soll 1 Jahr sein.

In den ersten Wochen und Monaten nach Transplantation kommt es besonders häufig zu Rejektions-Reaktionen und zu Infektionen, so dass in diesem Zeitraum die Erforschung innovativer Biomarker von besonderem Interesse ist. Gleichbedeutend damit ergibt sich für die Fragestellung (Definition von Referenzbereichen bzw. Beurteilung eines möglichen Nutzens) aus Messungen in den ersten Monaten eine besonders hohe statistische Power.

3. Ziele der Studie

In der Studie werden die Patienten wie bisher behandelt. Ergänzend zu den im Rahmen der Krankenversorgung regelmäßig durchgeführten Laboruntersuchungen einschließlich des therapeutischen Drug Monitoring werden im Rahmen der vorliegenden Studie die intrazellulären Konzentrationen der Immunsuppressiva sowie Biomarker der Immunaktivität gemessen. Darüber hinaus werden zirkulierende DNA differenziert nach Spender- und Empfänger quantifiziert als Maß für Organabstoßung oder Zellschädigung durch die Immunsuppressiva und es werden pharmakogenetische Parameter gemessen.

Vertrauliche Informationen: Weitergabe außerhalb der Studiendurchführung ohne Genehmigung der Autoren verboten.

Es ist das Ziel, aus der Auswertung dieser Daten in Relation zu den klinischen Daten der Patienten zu ermitteln, welche Parameter aussichtsreich sind, um davon abhängig in Zukunft die Auswahl und Dosierung von immunsuppressiven Medikamenten zu steuern.

Folgende spezifischen Teilziele sollen erreicht werden:

- Korrelation der intrazellulären Konzentrationen in den mononukleären Blutzellen mit den Konzentrationen im nicht fraktionierten Blut (so in der Regel für Cyclosporin und Tacrolimus verwendet) bzw. im Plasma (so in der Regel für Mycophenolsäure verwendet).
- Bestimmung therapeutischer Zielbereiche der intrazellulären Konzentrationen von Tacrolimus, Cyclosporin A, Mycophenolsäure und Everolimus (sofern eine Zulassung für die immunsuppressive Behandlung nach Lebertransplantationen in Deutschland erfolgt) in mononukleären Zellen des Blutes anhand der beiden klinischen Endpunkte Infektion und Rejektion und anhand ausgewählter Marker der Arzneimitteltoxizität.
- Analyse der Beziehung zwischen weiteren Nebenwirkungen der verwendeten Immunsuppressiva (Nierenfunktionsbeeinträchtigung, Hypercholesterinämie, diabetogene Wirkung, Diarrhoe, Leukopenie) in Relation zu den intrazellulären Arzneistoffkonzentrationen (itc-TDM) unter der Fragestellung, ob sich die Nebenwirkungen durch konsequente Anwendung des itc-TDM reduzieren lassen.
- Exploration des diagnostischen Wertes weiterer Biomarker der Immunzell-Aktivierung und der Immuntoleranz, um damit zu Indikatoren zu gelangen, die eine frühe Intervention bei sich andeutenden Krisen ermöglichen und andererseits eine minimal notwendige Immunsuppression erlauben zur Reduktion der Nebenwirkungen der Immunsuppression.
- Exploration des möglichen diagnostischen Wertes pharmakogenomischer Biomarker für die Individualisierung der immunsuppressiven Therapie. Dies betrifft zunächst (1.) Varianten mit Bedeutung für Gewebskonzentrationen (Pharmakokinetik) der Immunsuppressiva wie Varianten in den Genen der Arzneimittel-Membrantransportproteine (z.B. MDR1) und der Arzneimittel-metabolisierenden Enzyme (z.B. CYP3A4, CYP3A5). Das betrifft sodann (2.) Genvarianten in den Stoffwechselwegen und Signalwegen, die von den Immunsuppressiva beeinflusst werden und das betrifft schließlich (3.) Genvarianten in weiteren Proteinen, die für die Aktivität des Immunsystems von Bedeutung sind.
- Die Daten sollen die Grundlage für statistische Modelle liefern, denen in der Zukunft Entscheidungen bezüglich der Immunsuppressiven Therapie berechnet werden können. Dabei soll exploriert werden, welcher der genannten Biomarker Rejektionen oder Infektionen empfindlich voraussagen kann und es soll auch exploriert werden, ob Kombinationen der Biomarker besser als einzelne Biomarker die Rejektionen und Infektionen voraussagen können.

Soweit sich unsere Hypothesen bestätigen, könnte aus dem itc-TDM eine Reduktion an Transplantationskomplikationen und Arzneimittelnebenwirkungen resultieren, verbunden mit einer Verbesserung der Lebensqualität und der Lebenserwartung der Patienten. Sobald wir die geeigneten Biomarker identifiziert haben, erscheint des durchaus realistisch, Patienten zu identifizieren, bei denen eine Minimierung der Immunsuppression in Frage kommt.

Darüber hinaus möchten wir eine auf die Erforschung der Transplantations-Immunologie und Immunsuppression zweckbezogenen Biomaterialsammlung anlegen, um die Möglichkeit schaffen, auch weitere Biomarker zu evaluieren. In dieser besonderen Biobank zur Erforschung des Gesundheitsverlaufes nach Organtransplantationen sollen soweit möglich

Vertrauliche Informationen: Weitergabe außerhalb der Studiendurchführung ohne Genehmigung der Autoren verboten.

auch Blutproben bzw. DNA der Organspender gespeichert werden, da nur die Kombination aus beidem ein komplettes Bild vermitteln kann.

4. Studiendesign

4.1. Primäre und sekundäre Endpunkte

Primäre Endpunkte sind die intrazellulären Konzentrationen der Immunsuppressiva in den mononukleären Blutzellen (itc-TDM), deren Korrelation zu den nach gegenwärtiger medizinischer Praxis gemessenen TDM Konzentrationen analysiert werden soll.

Sekundäre Endpunkte sind Infektion (unter anderem Folge von Über-Immunsuppression) und Rejektion (unter anderem Folge von unzureichender Immunsuppression).

Infektion: Als Infektionen werden infektionstypische Verschlechterungen des Gesundheitszustandes gewertet, insbesondere wenn sie in der Verabreichung systemischer antiinfektiver Medikamente (Antibiotika, Antimykotika, Virustatika) resultieren. Todesfälle, die nach klinischen Daten oder Autopsie am ehesten als Folge von Infektionen (z.B. Sepsis, Pneumonie) erklärt werden können, werden dabei ebenfalls als Infektion gewertet, auch wenn keine Antiinfektiva verabreicht wurden.

Rejektion: Als Rejektion werden alle Verschlechterungen der Gesundheit bzw. der Organfunktion gewertet, die zu einer Rejektionstherapie (z.B. hochdosiert Glukokortikoide oder Biologicals) führen. Ein Todesfall, ein Verlust der Transplantatfunktion oder eine Re-Transplantation wird ebenfalls als Rejektion gewertet, wenn nach klinischer Einschätzung der behandelnden Ärzte und der beteiligten Pathologen die Rejektion die wahrscheinlichste Ursache ist.

Weitere sekundäre klinische Endpunkte sind alle klinischen und labormedizinischen Parameter, die Hinweise auf Unter- oder Über-Immunsuppression oder weitere Formen von Arzneimittel-Nebenwirkungen geben können. Dabei soll grundsätzlich die Erfassung auf das minimal Notwendige beschränkt werden und es werden ausschließlich diejenigen Parameter in die Studienunterlagen und Auswertung übernommen, die im Rahmen der klinischen Versorgung (also nicht forschungsbezogen) erfasst werden.

In der Folgenden Tabelle sind alle Parameter aufgeführt. Diejenigen, die zur Messung der primären Endpunkte beitragen, sind in Fettdruck hervorgehoben.

Parameter		Begründung
**itc-TDM**		**Primärer Endpunkt**
gGT	Gamma-Glutamyltransferase	Rejektion, Medikamenten-Hepatotoxizität (u.a. Cholestase)
ALAT	Alanin-Aminotransferase	Rejektion, Medikamenten-Hepatotoxizität
ASAT	Aspartat-Aminotransferase	Rejektion, Medikamenten-Hepatotoxizität
AP	Alkalische Phosphatase	Rejektion, Medikamenten-Hepatotoxizität (Cholestase)
Bilirubin	Gesamt-Bilirubin	Leberfunktion
Albumin		Leberfunktion
INR	*International normalized ratio*	Leberfunktion

Vertrauliche Informationen: Weitergabe außerhalb der Studiendurchführung ohne Genehmigung der Autoren verboten.

	(normierte Prothrombinzeit)	
Krea	Serum-Kreatininkonzentration	Nierenfunktion, Medikamenten-Nephrotoxizität
Leukozyten	Gesamt Leukozytenzahl im Blut	Infektion, Medikamenten-Hämatotoxizität
Lymphozyten	Gesamt Lymphozytenzahl im Blut	Infektion, Medikamenten-Hämatotoxizität
HbA1C	Glykosyliertes Hämoglobin	Medikamententoxizität
Gesamtcholesterin		Medikamententoxizität
LDL-Cholesterin		Medikamententoxizität (uptake durch Cyclosporin .gehemmt)
Triglyceride		Medikamententoxizität (VLDL, Insulin-Signaling)
Erfassung der Nebenwirkungen der immunsuppressiven Medikation	Abfrage zu den Visiten in der Ambulanz/Klinik	Medikamententoxizität
Blutdruck	Messung zu den Visiten in der Ambulanz/Klinik	Medikamententoxizität
Infektionen	Abfrage zu den Visiten in der Ambulanz/Klinik	Sekundärer Endpunkt
Rejektion	Biopsie	Sekundärer Endpunkt
Hospitalisation insgesamt	Krankenhaus-Aufenthaltstage insgesamt	Parameter der Lebensqualität
Hospitalisation immunsuppressionsbezogen	Krankenhaus-Aufenthaltstage bedingt durch Komplikationen der Immunsuppression	

4.2. Studiendesign

Es handelt sich um eine prospektive nicht-interventionelle Beobachtungsstudie (Kohortenstudie). Alle klinisch an den jeweiligen Zentren üblichen Therapien werden im Rahmen dieser Studie genauso wie zuvor durchgeführt. Die hier relevanten Details der Therapien werden dokumentiert.

Blutuntersuchungen erfolgen in den unten dargestellten Zeitintervallen nur in dem Rahmen, in dem auch außerhalb dieser Beobachtungstudie Kontrolluntersuchungen mit Venenpunktion bzw. Blutabnahmen erfolgen.

4.3. Maßnahmen gegen systematische Fehler bzw. Verzerrungen in den Ergebnissen

In Hinblick auf eine möglichst hohe Repräsentativität (externe Validität) sollen die Ein- und Ausschlusskriterien möglichst breit gehalten werden.

Anhand der im routinemäßigen therapeutischen Kontext abgenommenen Blutproben werden weitere innovative Biomarker analysiert. Der Voraussagewert dieser Biomarker hinsichtlich der primären und sekundären Endpunkte soll analysiert werden. Um Verfälschungen zu vermeiden, werden diese zusätzlich analysierten pharmakologischen und immunologischen bzw. zellbiologischen Marker weder für die Patientenversorgung zur Verfügung gestellt noch

Vertrauliche Informationen: Weitergabe außerhalb der Studiendurchführung ohne Genehmigung der Autoren verboten.

den dem Personal zur Einsicht zur Verfügung gestellt, das für die Dokumentation der klinischen Daten verantwortlich ist (Prinzip der Blindung).

4.4. Untersuchte Biomarker

In die Auswertung gehen alle gegenwärtig regelmäßig für die Therapieeinstellung berücksichtigten klinischen Marker und das therapeutische Drug Monitoring ebenso wie die innovativen hier speziell zu explorierenden Biomarker ein. In der folgenden Tabelle werden diese Biomarker aufgelistet mit einer kurze Begründung. Weitere Hintergründe sind in den einleitenden Kapiteln mit Zitate belegt dargestellt. Insgesamt bedeuten die zusätzlichen wissenschaftlich begründeten Blutabnahmen für den Patienten nur eine minimale Belastung und nur ein minimales Risiko, da die Blutabnahmen im Rahmen ohnehin erfolgender medizinisch indizierter Kontrolluntersuchungen stattfinden und da eine zusätzliche Entnahme von 20 ml Blut weder subjektiv noch empfindlich objektiv gemessen zu körperlichen Veränderungen führt.

Parameter (Kürzel, Parameter, Einheiten)		Begründung und ggf. weitere Erläuterungen	Dafür forschungsbedingt erforderliche Blutabnahme im Rahmen einer klinisch indizierten Visite
	Cyclosporin im Blut	Aus klinischen Routinemessungen in die Datenauswertungen übernommen	0 (da ohnehin erfolgend)
	Tacrolimus im Blut	Aus klinischen Routinemessungen in die Datenauswertungen übernommen	0 (da ohnehin erfolgend)
MPA	Mycophenolsäure im Plasma	Soweit durchgeführt, aus klinischen Routinemessungen in die Datenauswertungen übernommen, ansonsten zusätzlich gemessen	0 (da ohnehin erfolgend)
	(Everolimus* im Blut)	Soweit durchgeführt, aus klinischen Routinemessungen in die Datenauswertungen übernommen, ansonsten zusätzlich gemessen	0 (da ohnehin erfolgend)
Itc-CyA	Intrazelluläres Cyclosporin	Primärer zu untersuchender Biomarker bei Cyclosporin-Therapie	6 ml
Itc-Tacro	Intrazelluläres Tacrolimus	Primärer zu untersuchender Biomarker bei Tacrolimus-Therapie	
Itc-MPA	Intrazelluläre Mycophenolsäure	Primärer zu untersuchender Biomarker bei Mycophenolsäure-Therapie	
(Itc-Everolimus*)	(Intrazelluläres Everolimus)	Primärer zu untersuchender Biomarker bei Everolimus-Therapie	

Vertrauliche Informationen: Weitergabe außerhalb der Studiendurchführung ohne Genehmigung der Autoren verboten.

CD4+ Immun-antwort	ng/ml ATP in PHA-stimulierten CD4+ T-Zellen	Immunzell-Aktivitätsmarker	3 ml
IL-2/CD8+	Anteil IL-2+ T-Zellen der gesamten CD8+ T-Zellen, Angabe in Prozent	Immunzell-Aktivitätsmarker	3 ml
IGKV1D-13, IGKV4-1 und IGLL1	Expression der toleranz-relevanten B-Zell-Differenzierungsgene , normalisierte mRNA-Kopienzahl	Immuntoleranzmarker	3 ml
FoxP3	Als FACS-Messung	Immuntoleranzmarker	3 ml
FoxP3	Als DNA-Methylsierungsmessung	Immuntoleranzmarker	2 ml
CNA	Im Plasma zirkulierende DNA	Marker zur Differentialdiagnose von Rejektion versus anderen Organschäden	0 (aus Überstand bei Zellisolierungen)
			Insgesamt 20 ml Blut forschungsbedingt

* sofern und erst nach Zulassung für die immunsuppressive Therapie in Deutschland

Pharmakogenetische Biomarker

In der DNA des Patienten sollen sowohl Kandidatengen-bezogen als auch im Rahmen eines hypothesenfreien Screening Genvarianten mit Bedeutung für Medikamentenwirkungen und Nebenwirkungen sowie mit Bedeutung für Rejektionsrisiko und Infektionsrisiko untersucht werden.

In der DNA des transplantierten Organs sollen ebenfalls sowohl Kandidatengen-bezogen als auch im Rahmen eines hypothesenfreien Screening Genvarianten mit Bedeutung für Medikamentenwirkungen und Nebenwirkungen sowie mit Bedeutung für Rejektionsrisiko untersucht werden.

In einigen Fällen (unklare Nebenwirkungen oder schlecht erklärbare Rejektionen) kann individuell medizinisch die Messung einer Blutkonzentration zu 2 Stunden nach Dosierung (C2) oder die Dokumentation eines Tagesprofils der Blutkonzentrationen der Arzneimittel angebracht sein. Sofern derartige Messungen bei in die Studie eingeschlossenen Patienten erfolgen, werden hier ebenfalls auch die intrazellulären Konzentrationen erfasst.

4.5. Studiendauer und Zeitplan

Die Studie insgesamt soll sobald möglich beginnen und nach Studienbeginn als Studiendauer 18 Monate dauern. Für den einzelnen Patienten soll die Beobachtungsdauer 1 Jahr betragen.

Für den einzelnen Patienten ergeben sich die Studienzeitpunkte aus den klinisch üblichen Visiten und Krankenhausaufenthalten. Darüber hinaus kommt es bei allen möglichen Komplikationen (z.B. Rejektion oder Infektion) zu zusätzlichen Visiten. Beides, die regulären Visiten im Rahmen der Nachkontrolle und die Visiten bzw. Hospitalisierungen bei

Vertrauliche Informationen: Weitergabe außerhalb der Studiendurchführung ohne Genehmigung der Autoren verboten.

Komplikationen erfolgen nicht studienbedingt. Entsprechend dient der folgende Zeitplan nur zur Orientierung hinsichtlich der maximalen Anzahl zusätzlicher Laboranalysen.

Nr.	Stichtag nach LTx*	Zeitraum nach LTx*	TDM (Blut, Plasma)**	Itc-TDM (intrazellulär)	CD4-Immunantwort	IL-2/CD8	FoxP3	B-Zell-Differenzierungsgene	CNA (circulierende DNA)	Klin-Chemische Analysen**	Klinische Daten**	Aufzeichnung von Events**	Pharmakogenomik
0		Einmalig irgendwann											X
1	1 Woche	0 – 10 Tage	X	X		X	X	X	X	X	X	X	
2	2 Wochen	11 – 30 Tage	X	X	X	X	X	X	X	X	X	X	
3	6 Wochen	1 – 2 Monate	X	X		X	X	X	X	X	X	X	
4	3 Monate	2 – 4 Monate	X	X		X	X	X	X	X	X	X	
5	6 Monate	4 – 8 Monate	X	X	X	X	X	X	X	X	X	X	
6	9 Monate	8 – 10 Monate	X	X		X	X	X	X	X	X	X	
7	12 Monate	10 – 14 Monate	X	X	X	X	X	X	X	X	X	X	
Im Rahmen akuter Komplikationen (Verdacht auf oder bestätigte akute Rejektionen und/oder Infektionen)***			x	x	X	X			X	X	X	X	
Seriell zur Verlaufsdokumentation***			x	x	X	X			X	X	X	X	

* Alle genannten Zeitpunkte entfallen wenn die Patienten in diesem Zeitraum nicht in die ambulante Nachbetreuung kommen.

** Diese Messungen oder Erhebungen werden im Rahmen der Beobachtungsstudie ausgewertet, aber nicht studienbedingt durchgeführt.

*** Diese Messungen werden durchgeführt, um die Parameter besser einschätzen zu können. Wir sind uns dabei dessen bewusst, dass Biomarker, die im Rahmen akuter Störungen gemessen werden, sowohl Ursache als auch Folge der Störung sein können. Abweichungen bei Biomarkern gemessen bei akuten Störungen bedeuten damit nicht, dass dieses Biomarker einen prädiktiven Wert für akute Störungen haben.

4.6. Abbruchkriterien

Die Beobachtungsstudie geht nur mit minimalen Risiken einher, insofern sind hier keine Abbruchkriterien für die Untersuchung des einzelnen Studienteilnehmers bzw. der Studie

Vertrauliche Informationen: Weitergabe außerhalb der Studiendurchführung ohne Genehmigung der Autoren verboten.

insgesamt vorgesehen. Die unabhängig davon gültigen Gründe für eine Beendigung der Studie am individuellen Patienten sind weiter unten dargestellt. Ein Abbruch der Studie insgesamt bzw. einem der Studienzentrum aus finanziellen oder organisatorischen Gründen ist sehr unwahrscheinlich, kann aber auch nicht restlos ausgeschlossen werden.

4.7. Datenmanagement

Alle Daten dieser Studie werden in GCP-kompatibler Form auf Papier erfasst bzw. in entsprechend validierte Datenbanken eingegeben. Soweit Daten in das bekanntlich nicht GCP-kompatible Computerprogramm Microsoft-Excel eingegeben werden, werden die entsprechenden Datenblätter zeitnahe ausgedruckt und abgezeichnet und im Studienordner gelagert.

Für die Studie wird eine GCP-kompatible Datenbank angelegt, in die alle Daten pseudonymisiert über einen Internet-Browser eingegeben werden.

5. Auswahl und Ausschluß von Studienteilnehmern

5.1. Einschlußkriterien

Einwilligungsfähigen Patienten und Patientinnen innerhalb der ersten 24 Monate nach Lebertransplantation

5.2. Ausschlußkriterien

Jede Beteiligung an Therapiestudien zu anderen immunsuppressiven Medikamenten, es sei denn, dass der Teilnahme an vorliegender Beobachtungsstudie vom jeweiligen Sponsor schriftlich zugestimmt wurde.

Fehlende Einwilligungsfähigkeit
Alter < 18 Jahre

5.3. Kriterien für individuellen Studienabbruch und Dokumentation in diesen Fällen

- Abbruch auf Wunsch des individuellen Patienten (nach Möglichkeit sollen die Gründe erfasst werden)
- Abbruch durch Wegzug des Patienten aus dem Einzugsbereich des Studienzentrums
- Abbruch durch Notwendigkeit einer Re-Transplantation
- Entscheidung des Studienzentrums, den Patienten in eine Forschungsstudie zu einem neuen immunsuppressiven Medikament aufzunehmen

6. Arzneitherapie und weitere Behandlung

Im Rahme der Studie wird kein Einfluss auf die Medikation genommen. Innerhalb des Erfassungszeitraumes wird die immunsuppressive Medikation so vollständig wie möglich dokumentiert.

Vertrauliche Informationen: Weitergabe außerhalb der Studiendurchführung ohne Genehmigung der Autoren verboten.

Ebenso werden die antiinfektive Medikation (Antibiotika, Virusstatika, Antimykotika) sowie die antihypertensive, antidiabetische und cholesterinsenkende Therapie als sekundäre Endpunkte dokumentiert.

7. Statistik

7.1. Statistische Datenauswertung

Zunächst werden alle Meßwerte der Biomarker in deskriptiver Statistik für die gesamte Stichprobe und für die Untergruppen mit Rejektion innerhalb von 2 Wochen nach Meßwert bzw. Infektion innerhalb von 2 Wochen nach Meßwert dargestellt. Die weitergehende Evaluation der Biomarker wird auf Multivariate Mixed Models beruhen, wozu es in der dafür verantwortlichen Abteilung Medizinische Statistik (Direktor: Prof. Dr. Tim Friede) langjährige Erfahrungen gibt. Darüber hinaus werden pharmakokinetisch-pharmakodynamische Modellanalysen durchgeführt mit dem Ziel zu explorieren, inwieweit man in Zukunft vielleicht auf den Biomarkern basierende berechnete Dosierungsempfehlungen geben könnte. Das soll natürlich keinesfalls in gegenwärtiger Studie in die Therapie einfließen, derartiges wird aber in der langfristigen Perspektive sicher auch zu einer personalisierten Medizin gehören.

7.2. Begründung für Fallzahl

Wir möchten die Studie an 125 PatiententInnen durchführen, die über 1 Jahr nachverfolgt werden können. Wir gehen davon aus, dass etwa 20% aus unterschiedlichen Gründen zu früh aus der Studie ausscheiden und insofern nicht auswertbar sein werden (*drop out*) so dass wir wahrscheinlich Initial 150 Patienten einschließen werden.

Die Analyse der intrazellulären Zielkonzentrationen der Medikamente basiert auf der Korrelation der intrazellulären Zielkonzentrationen mit den heute üblicherweise gemessenen Gesamtblutkonzentrationen. Dabei wäre ein Stichprobenumfang von 115 Patienten ausreichend um bei einem Korrelationskoeffizienten (Pearson) von mindestens 0,3 zu einer hinreichend präzisen Bestimmung der intrazellulären Zielkonzentrationen zu kommen.

Bei einer Häufigkeit von Infektionen und Rejektionen im ersten Jahr nach Transplantation von ca. 30% wird es unter den verbleibenden 125 Patienten ca. 40 Rejektionen und ca. 40 Infektionen geben. Daraus ergibt sich, dass die vorgesehene Zahl von Patienten wahrscheinlich ausreichend ist, um basierend auf den genannten klinischen Endpunkten die Eignung und die therapeutischen Bereiche der unterschiedlichen Biomarker abschätzen zu können.

Alle weiteren Analysen werden als sekundär betrachtet und im Rahmen der tatsächlichen Fallzahlen bewertet.

7.3. Kriterien für Studienabbruch

Grundsätzliche medizinische oder wissenschaftliche Gründe, die zu einem Studienabbruch zwingen würden, sind kaum denkbar. Insofern gibt es hier keine vordefinierten Kriterien für einen Studienabbruch.

7.4. Umgang mit fehlenden und unvollständigen Daten

Vertrauliche Informationen: Weitergabe außerhalb der Studiendurchführung ohne Genehmigung der Autoren verboten.

Wir gehen davon aus, dass nur sehr wenige Patienten die Teilnahme an der Studie abbrechen werden, da keine wesentlichen Belastungen aus der Studie resultieren bzw. medizinische Kontrollen ohnehin unabhängig von der Studie nötig sind. Unabhängig davon sind die in der Auswertung verwendeten Multivariate Mixed Models recht robust gegenüber fehlenden Werten (Molenberghs G, Kenward MG (2007) Missing Data in Clinical Studies; Chapter 7). Sollte wider Erwarten hohe Anteile an Dropouts auftreten, so würden wir auch hier unter Verwendung der oben angegebenen Verfahren die Hypothese des unabhängigen Dropouts untersuchen.

7.5. Änderungen am statistischen Auswertekonzept

Alle Änderungen werden dokumentiert, begründet und als administratives Amendement der zuständigen Ethik-Kommission mitgeteilt

7.6. Kriterien für Ausschluss von Daten und Patienten von der statistischen Auswertung und Publikation

Messungen, deren Werte mehr als 3 Standardabweichungen nach oben oder nach unten vom Erwartungswert abweichen, werden nach Möglichkeit wiederholt. Ein Ausschluß von Werten bei der Analyse erfolgt nur in den Fällen, in denen nach schriftlich dokumentierter und begründeter Einschätzung von 2 Experten der Meßwert medizinisch hochgradig unwahrscheinlich ist. Nicht gewertet und durch einen weiteren Patienten ersetzt wird jeder Patient, bei dem weniger als 3 Meßzeitpunkte vorliegen, es sei denn, dass das Ende durch einen klinischen Endpunkt (Infektion oder Rejektion) bedingt ist und der Patient nicht weiter in der Studie verfolgt werden kann, weil der Patient entweder stirbt, oder eine Re-Transplantation oder eine prinzipiell andere Immunsuppression, die den Einschlusskriterien nicht mehr gehorcht, erforderlich werden.

8. Zugang zu Quelldaten

In Hinblick auf die Datenqualität stimmen die Studienzentren und Studienpatienten der Einsicht von StudienmitarbeiterInnen in die Originaldaten zu. Zu dieser Einsicht ist nur autorisiertes Studienpersonal berechtigt, dass über entsprechende Ausbildung verfügt, das den jeweiligen Studienzentren namentlich bekannt ist (Delegationsliste) und das, sofern nicht an der Institution angestellt, nachweislich über Vertraulichkeit im Umgang mit Patientendaten belehrt wurde und dazu eine Datenschutzerklärung unterschrieben hat. Die Patienten stimmen auch zu, dass bei zu Fragen in Zusammenhang mit dieser Studie auch Informationen bei mitbehandelnden Ärzten angefordert werden, wenn dieses nötig sein sollte.

9. Maßnahmen zur Sicherung der Datenqualität

An allen Studienzentren findet ein regelmäßiges Monitoring statt. Hierzu wird ein Monitoringplan erstellt. Alle Daten sind schriftlich in den Originalunterlagen dokumentiert und werden in einer validierte Datenbank mit *audit trail* eingegeben.

Vertrauliche Informationen: Weitergabe außerhalb der Studiendurchführung ohne Genehmigung der Autoren verboten.

10. Ethische und rechtliche Gesichtspunkte

Im Rahmen dieser Studie finden keine studienbedingten Interventionen in die Arzneitherapie statt. Die Blutentnahme für die zusätzlichen hier interessierenden innovativen Biomarker erfolgt im Rahmen regelmäßiger Kontrolluntersuchungen, so dass keine studienbedingten Venenpunktionen erforderlich sind.

Pro Visite werden zur Messung der molekularen Biomarker 20 ml Blut entnommen. Dies erfolgt in Abständen, die in der Regel größer als 1 Monat sind. Separate nur studienbedingte Punktionen erfolgen nicht. Wir denken, dass diese zusätzlichen Blutmengen nur mit einem minimalen Risiko und nur mit einer minimalen Belastung einhergehen.

Einer gewissen Belästigung der Studienteilnehmer durch Datenerfassung stehen Chancen für eine deutlich verbesserte Therapieeinstellung von Patienten aus dieser Gruppe gegenüber. Auch die Patienten selbst können davon profitieren.

Im Bezug auf das Arzneimittelgesetz handelt es sich um eine nicht-interventionelle Studie. Dabei ist das Ziel der Studie nicht die Erforschung von Metabolismus, Pharmakokinetik, Wirkungen oder Nebenwirkungen der Medikamente, denn diese Fakten sind hinreichend bekannt. Diese Daten werden hier aber benötigt, um den möglichen klinischen Wert der neuen Verfahren des therapeutischen Drug Monitoring zu bestimmen.

Im Bezug auf das Medizinproduktegesetz findet hier keine Erprobung neuer in-vitro Diagnostika statt. Es werden für Blutabnahmen selbstverständlich ausschließlich entsprechend zertifizierte Produkte verwendet. Langfristig ist denkbar, dass wir, je nach Erfolg der hier zu prüfenden Parameter zur Erleichterung der praktischen Durchführung die Entwicklung entsprechender neuartiger in-vitro Diagnostika anstoßen werden. Im Rahmen der vorliegenden Studie ist die Entwicklung neuer in-vitro-Diagnostika aber nicht geplant. Alle Laboranalysen werden in einem für die klinisch-chemische Labordiagnostik zertifizierten Labor in GCP-konformer Weise durchgeführt.

11. Datenmanagement

Alle Daten werden in Papierform dokumentiert und in einer GCP-kompatiblen Datenbank gespeichert.

12. Finanzierung der Studie und Versicherung

Die Studie wird finanziert durch die Projektförderung seitens des Bundesministeriums für Bildung und Forschung (Förderkennzeichen 01ES1102).

Da die Studie nur im Rahmen ohnehin aus klinischem Kontext stattfindender Visiten und Blut abnahmen erfolgt, sind die gesundheitlichen Risiken durch die Studie minimal. Es wird keine Patientenversicherung abgeschlossen.

13. Publikationsvereinbarungen

Alle wissenschaftlichen Daten aus der Studie werden gemeinsam nach Erreichen der geplanten Fallzahlen publiziert.

Vertrauliche Informationen: Weitergabe außerhalb der Studiendurchführung ohne Genehmigung der Autoren verboten.

Alle Fragen zur Publikation werden in einem Publikationskommittee persönlich oder im e-mail Umlauf beraten. Dem Publikationskommittee gehören mindestens folgende Personen an: Je Studienzentrum mindestens ein Arzt oder eine Ärztin sowie die Professoren Oellerich, Friede und Brockmöller. Weitere Personen können nach Absprache in das Publikations-kommittee aufgenommen werden.

Individuelle Veröffentlichungen einzelner Studienaspekte vor der jeweiligen abschließenden Publikation ebenso wie die Veröffentlichung von Promotionsarbeiten werden ausdrücklich auch durch Bereitstellung entsprechender Daten aus der Datenbank bzw. aus den Labor-untersuchungen unterstützt.

Bezüglich Promotionsarbeiten soll aber mit dem/der jeweiligen Doktoranden/in geklärt werden, dass die Veröffentlichung der Doktorarbeit gegebenenfalls bis zu einer Publikation in einer wissenschaftlichen Fachzeitschaft verzögert werden muss.

14. Zitierte Literatur

Boleslawski, E., F. Conti, et al. (2004). "Defective inhibition of peripheral CD8+ T cell IL-2 production by anti-calcineurin drugs during acute liver allograft rejection." Transplantation **77**(12): 1815-20.

Compagni, A., S. Bartoli, et al. (2008). "Avoiding adverse drug reactions by pharmacogenetic testing: a systematic review of the economic evidence in the case of TPMT and AZA-induced side effects." Int J Technol Assess Health Care **24**(3): 294-302.

Crettol, S., J. P. Venetz, et al. (2008). "Influence of ABCB1 genetic polymorphisms on cyclosporine intracellular concentration in transplant recipients." Pharmacogenet Genomics **18**(4): 307-15.

Falck, P., A. Asberg, et al. (2008). "Declining intracellular T-lymphocyte concentration of cyclosporine a precedes acute rejection in kidney transplant recipients." Transplantation **85**(2): 179-84.

Holt, D. W., V. W. Armstrong, et al. (2002). "International Federation of Clinical Chemistry/International Association of Therapeutic Drug Monitoring and Clinical Toxicology working group on immunosuppressive drug monitoring." Ther Drug Monit **24**(1): 59-67.

Kamdem, L. K., F. Streit, et al. (2005). "Contribution of CYP3A5 to the in vitro hepatic clearance of tacrolimus." Clin Chem **51**(8): 1374-81.

Kowalski, R., D. Post, et al. (2003). "Immune cell function testing: an adjunct to therapeutic drug monitoring in transplant patient management." Clin Transplant **17**(2): 77-88.

Millan, O., C. Benitez, et al. (2010). "Biomarkers of immunoregulatory status in stable liver transplant recipients undergoing weaning of immunosuppressive therapy." Clin Immunol **137**(3): 337-46.

Newell, K. A., A. Asare, et al. (2010). "Identification of a B cell signature associated with renal transplant tolerance in humans." J Clin Invest **120**(6): 1836-47.

Pons, J. A., B. Revilla-Nuin, et al. (2008). "FoxP3 in peripheral blood is associated with

Vertrauliche Informationen: Weitergabe außerhalb der Studiendurchführung ohne Genehmigung der Autoren verboten.

operational tolerance in liver transplant patients during immunosuppression withdrawal." Transplantation **86**(10): 1370-8.

Schulz-Juergensen, S., M. M. Burdelski, et al. (2012). "Intracellular ATP production in CD4+ T cells as a predictor for infection and allograft rejection in trough-level guided pediatric liver transplant recipients under calcineurin-inhibitor therapy." Ther Drug Monit **34**(1): 4-10.

Schütz, E., J. Gummert, et al. (1995). "Azathioprine myelotoxicity related to elevated 6-thioguanine nucleotides in heart transplantation." Transplant Proc **27**(1): 1298-300.

Shaw, L. M., B. Kaplan, et al. (1998). "Prospective investigations of concentration-clinical response for immunosuppressive drugs provide the scientific basis for therapeutic drug monitoring." Clin Chem **44**(2): 381-7.

Snyder, T. M., K. K. Khush, et al. (2011). "Universal noninvasive detection of solid organ transplant rejection." Proc Natl Acad Sci U S A **108**(15): 6229-34.

Touw, D. J., C. Neef, et al. (2005). "Cost-effectiveness of therapeutic drug monitoring: a systematic review." Ther Drug Monit **27**(1): 10-7.

Wieczorek, G., A. Asemissen, et al. (2009). "Quantitative DNA methylation analysis of FOXP3 as a new method for counting regulatory T cells in peripheral blood and solid tissue." Cancer Res **69**(2): 599-608.

Vertrauliche Informationen: Weitergabe außerhalb der Studiendurchführung ohne Genehmigung der Autoren verboten.
