# Supplementary material for: Graft-derived cell-free DNA, a noninvasive early rejection and graft damage marker in liver transplantation: A prospective, observational, multicenter cohort study
Source: PLoS Med. 2017 Apr 25;14(4):e1002286. doi: 10.1371/journal.pmed.1002286 (PMC5404754; doi:10.1371/journal.pmed.1002286)
Supplement: S2 Text — (PDF) [file pmed.1002286.s015.pdf]

Personalized Immunosuppression after Liver Transplantation: An observational study on the use and therapeutic area of innovative biomarkers

Prof. Dr. med. Otto Kollmar
Clinic for General and Visceral Surgery
University Medical Center Göttingen
Robert-Koch-Str. 40
Tel: +49 551 39 2260
Fax: +49 551 39 2202
otto.kollmar@med.uni-goettingen.de

Dr. med. Jochen Gaedcke
Clinic for General and Visceral Surgery
University Medical Center Göttingen
Robert-Koch-Str. 40
Tel: +49 551 39 6944
Fax: +49 551 39 12550
jochen.gaedcke@med.uni-goettingen.de

Laboratory analyses:
Herr Prof. Dr. Dr. med. Michael Oellerich
Department of Clinical Chemistry, University Medical Center Göttingen,
Robert-Koch-Str. 40, 37075 Göttingen
Tel: +49 551 39 8561
Fax: +49 551 39 8551 (zentrales Studienfaxgerät: 0551 39 22194)
michael.oellerich@med.uni-goettingen.de

Study coordination and monitoring:
Prof. Dr. med. Jürgen Brockmöller
Department of Clinical Pharmacology, University Medical Center Göttingen,
Robert-Koch-Str. 40, 37075 Göttingen
Tel: +49 551 39 5770
Fax: +49 551 39 12767
jbrockm@gwdg.de

Table of Contents

1. General information 5
1.1. Study title and protocol versions 5
1.2. Sponsor and monitoring 5
1.3. Responsible physicians and scientists 5
1.4. Medical and Scientific Advisory Council 7
2. Introduction: Medical and scientific background 7
2.1. Immunosuppression after liver transplantation 7
2.2. Current status of therapeutic drug-concentration monitoring (TDM) after liver transplantation 8
2.3. Current state of therapeutic immune monitoring after liver transplantation 8
2.4. Current status of pharmacogenetic testing in immunosuppression 10
2.5. Further information about the patient group examined here 10
3. Objectives of the study 10
4. Study design 11
4.1. Primary and secondary endpoints 11
4.2. Study design 13
4.3. Measures to guard against systematic errors or distortions of the results 13
4.4. Examination of biomarkers 13
4.5. Study duration and schedule 15
4.6. Cancellation criteria 16
4.7. Data management 16
5. Selection and exclusion of study participants 16
5.1. Inclusion criteria 16
5.2. Exclusion criteria 16
5.3. Criteria for individual study termination and documentation in such cases 16
6. Drug therapy and further treatment 17
7. Statistics 17
7.1. Statistical data evaluation 17
7.2. Sample size justification 17
7.3. Criteria for discontinuation of studies 17
7.4. Handling of missing and incomplete data 18
7.5. Changes to the planned statistical evaluation 18
7.6. Criteria for exclusion of data and patients from statistical evaluation and publication 18
8. Access to source data 18
9. Measures to ensure data quality 18
10. Ethical and legal aspects 19
11. Data management 19
12. Study Financing and insurance 19
13. Publication agreements 19
14. Supporting Literature 20

Synopsis

**Title**	**Personalized immunosuppression after liver transplantation: An observation study on the use and therapeutic area of innovative biomarkers**<br><br>(Biomarker assisted personalized immunosuppression following liver transplantation)
**Study design**	Prospective non-interventional observation study (cohort study)
**Background**	Immunosuppressive therapy after transplantation represents a balancing act between infection (overdosing) on one side and rejection (underdosing) on the other side.<br><br>The uptake of many immunosuppressants into the relevant immune cells and the excretion from the cells is very variable. Therefore, the measurement of intracellular target concentrations of immunosuppressants in the future is likely to lead to much better individual therapy adaptation than currently practiced forms of therapeutic drug monitoring.<br><br>We assume that there are also individual differences in immunosuppression, even with identical blood and tissue levels. Therefore, the measurement of further immune biomarkers in the future will probably allow for better individual therapy adjustment.
**Study funding**	The study is funded by the German Federal Ministry of Education and Research (ID number 01ES1102).
**Study objectives**	In the context of the medically indicated therapy, data analyzes and supplementary laboratory analyzes in a relatively large number of patients after liver transplantation will contribute to the following objectives.<br>• Correlation of the intracellular concentrations in mononuclear blood cells (itc-TDM) with the concentrations in the non-fractionated blood (TDM) as well as definition of therapeutic target ranges for intracellular concentrations of immunosuppressants.<br>• Exploration of innovative biomarkers for immune cell activation monitoring (especially IL2-CD8 and CD4 activation) and immune tolerance monitoring (especially FOXP3 and B-cell differentiation genes) as well as other cytokines detectable in the blood plasma.<br>• Measurement of plasma circulating DNA (CNA) differentiated according to DNA from organ donor and recipient regarding the possible diagnostic value in the early detection and differential diagnosis of rejections.<br>• Exploration of the potential diagnostic value of pharmacogenomic biomarkers for the individualization of immunosuppressive therapy.<br>The reference areas identified in the present study and the markers that are found to be informative should, after confirmation in a follow-up study, help to make the immunosuppression of patients after liver transplantation more effective which should, in particular, produce fewer side effects and therefore make it safer.
**Endpoints**	• Primary endpoint: Measurement of the intracellular concentrations in the mononuclear blood cells (itc-TDM) and their correlation to the concentrations in the non-fractionated blood (TDM)<br>• Secondary endpoints: infections and rejections after liver transplantation as well as clinical and laboratory parameters indicating signs of under or over-suppression and side effects of medications
**Patient numbers and duration**	• 125 patients after liver transplantation<br>• Locations: University of Göttingen, Department of General and Visceral Surgery, University Hospital Hamburg Eppendorf, Clinic and Polyclinic for Hepatobiliary Surgery and Transplantation Surgery and Charité Berlin, Campus

	Virchow-Klinikum, Department of General, Visceral and Transplantation Surgery<br>• Study duration: 18 months with an observation period per patient of 12 months
**Inclusion criteria**	• Consented patients within the first 9 months after liver transplantation<br>• Treatment with at least one of the medications: cyclosporin A, tacrolimus, mycophenolic acid, and everolimus (the latter only if and after an authorization for this therapy after liver transplantation exists in Germany)
**Exclusion criteria**	• Receiving therapy with other immunosuppressive drugs (exception: sponsor agrees to enroll)<br>• Lack of consent<br>• Age <18 years
**Statistical data evaluation**	Prof. Dr. Tim Friede<br>Department of Medical Statistics<br>University Medical Center Göttingen<br>Humboldallee 32<br>37075 Göttingen<br>Tel: +49 551 39 4990<br>Fax: +49 551 39 4995<br>tim.friede@med.uni-goettingen.de
**Management**	Prof. Dr. med. Otto Kollmar<br>Clinic for General and Visceral Surgery<br>University Medical Center Göttingen<br>Robert-Koch-Str. 40<br>Tel: +49 551 39 2260<br>Fax: +49 551 39 2202<br>otto.kollmar@med.uni-goettingen.de<br><br>Dr. med. Jochen Gaedcke<br>Clinic for General and Visceral Surgery<br>University Medical Center Göttingen<br>Robert-Koch-Str. 40<br>Tel: +49 551 39 6944<br>Fax: +49 551 39 12550<br>jochen.gaedcke@med.uni-goettingen.de<br><br>Laboratory analyses:<br>Herr Prof. Dr. Dr. med. Michael Oellerich<br>Department of Clinical Chemistry<br>niversity Medical Center Göttingen<br>Robert-Koch-Str. 40<br>37075 Göttingen<br>Tel: +49 551 39 8561<br>Fax: +49 551 39 8551<br>michael.oellerich@med.uni-goettingen.de<br><br>Responsible for study coordination and monitoring:<br>Prof. Dr. med. Jürgen Brockmöller<br>Department of Clinical Pharmacology<br>University Medical Center Göttingen<br>Robert-Koch-Str. 40<br>37075 Göttingen<br>Tel: +49 551 39 5770<br>Fax: +49 551 39 12767<br>jbrockm@gwdg.de

1. General information

1.1. Study title and protocol versions

Personalized immunosuppression after liver transplantation:
An observation study of the use and therapeutic area of innovative biomarkers

Acronym: PI-LTX

Protocol version: 1.0

1.2. Sponsor and monitoring

This is a multi center, academic investigator initiated, and non-interventional observational study of immunosuppression monitoring after liver transplantation.

The implementation of the study is assured by the support of the Federal Ministry of Education and Research (ID number 01ES1102) and through the additional commitments of the involved medical-scientific institutions.

The monitoring will be carried out by an appropriately trained scientist from the Department of Clinical Pharmacology of the University Medical Center Göttingen.

1.3. Responsible physicians and scientists

Prof. Dr. med. Otto Kollmar
Clinic for General and Visceral Surgery
University Medical Center Göttingen
Robert-Koch-Str. 40
Tel: +49 551 39 2260
Fax: +49 551 39 2202
otto.kollmar@med.uni-goettingen.de

Dr. med. Jochen Gaedcke
Clinic for General and Visceral Surgery
University Medical Center Göttingen
Robert-Koch-Str. 40
Tel: +49 551 39 6944
Fax: +49 551 39 12550
jochen.gaedcke@med.uni-goettingen.de

In addition to the persons mentioned here, additional persons will participate in this observational study. These will appear on a delegation list kept at the respective centers with their respective rights, tasks and obligations recorded in writing.

<u>Those responsible for the multi-center study coordination and monitoring are:</u>

Herr Prof. Dr. med. Jürgen Brockmöller
Department of Clinical Pharmacology
University Medical Center Göttingen
Robert-Koch-Str. 40
37075 Göttingen
Tel: +49 551 39 5770
Fax: +49 551 39 12767
jbrockm@gwdg.de

Laboratory analyses
Scientific coordination:
Herr Prof. Dr. Dr. med. Michael Oellerich
Department of Clinical Chemistry
University Medical Center Göttingen
Robert-Koch-Str. 40
37075 Göttingen
Tel: +49 551 39 8561
Fax: +49 551 39 8551
michael.oellerich@med.uni-goettingen.de

Within the Department of Clinical Chemistry: Molecular Immunological Analysis:
Herr PD Dr. med. Gunnar Brandhorst
Department of Clinical Chemistry
University Medical Center Göttingen
Robert-Koch-Str. 40
37075 Göttingen
Tel: +49 551 39 8062
Fax: +49 551 39 8551
gunnar.brandhorst@med.uni-goettingen.de

Within the Department of Clinical Chemistry: Drug Concentration Analyses:
Herr Dr. rer. nat. Frank Streit
Department of Clinical Chemistry
University Medical Center Göttingen
Robert-Koch-Str. 40
37075 Göttingen
Tel: +49 551 39 5792
Fax: +49 551 39 8551
fstreit@med.uni-goettingen.de

Statistical planning and evaluation:
Herr Prof. Dr. Tim Friede
Department of Medical Statistics
University Medical Center Göttingen
Humboldtallee 32
37073 Göttingen
Tel: +49 551 39 4990
Fax: +49 551 39 4995
tim.friede@med.uni-goettingen.de

1.4. Medical and Scientific Advisory Council

The course of studies and all protocol changes will be agreed to by the medical and scientific advisory board. The medical and scientific advisory council is listed in items 1.1. To 1.4. Other persons may be included in this Advisory Board after unanimous agreement.

An independent data safety monitoring board is not required for this observational study, which involves only minimal risks and burdens.

2. Introduction: Medical and scientific background

2.1. Immunosuppression after liver transplantation

As a rule, after a liver transplant, patients require long-term basic immunosuppression. Within the framework of basic immunosuppression, combinations of two or three of the following medications are typically used: a) a so-called calcineurin inhibitor (cyclosporin or tacrolimus), b) a glucocorticoid (prednisone or methylprednisolone), and c) an antimetabolite Mycophenolate mofetil or azathioprine). The subject of the present observation study is exclusively therapies with immunosuppressants which are already approved for use in the liver transplantation.

Immediately before and during the weeks after transplantation, as well as during any acute organ rejection (rejections) additional, very strong immunosuppressive drugs are used for induction and rejection therapy.

With basic immunosuppression organ rejection can usually be prevented. However, this immunosuppressive therapy involves a tight balancing act between under dosing with the risks of organ rejection on the one hand and overdosing with the risks of possibly life-threatening infections and dose-dependent drug side effects.

There is extensive clinical experience in the selection and dosage of optimal individual immunosuppression. Nevertheless, individual responses to immunosuppressive drugs is predictable to only a limited extent, and even in the same patient the need for immunosuppressants can fluctuate significantly over the months and years after transplantation. Reliable, medically accepted ways to determine in medical practice whether a patient is properly immunosuppressed at any given time, that is, neither under-treated nor over-treated, do not currently exist.

There are some reliable, so-called clinical endpoints for rejection and infection as well as for some side effects of immunosuppressant drugs. However, one would not like to have more than these so-called clinical endpoints, as these may be difficult to use or may impair the quality of life of the patients due to the need for intensive treatments and hospitalizations. Therefore so-called surrogate parameters are used, these are auxiliary variables, which can give important partial hints as to whether a patient is receiving the right dose or the right choice of medications. Long-established surrogate parameters include laboratory markers of organ function and therapeutic drug monitoring (drug concentration monitoring).

2.2. Current status of therapeutic drug-concentration monitoring (TDM) after liver transplantation

The individual selection of the dose of drugs such as cyclosporin, tacrolimus, or everolimus is guided by measurement of blood or plasma concentrations (therapeutic drug monitoring). This is a therapeutic standard in transplantation medicine (Holt et al. 2002) and is also disclosed in corresponding specialist information, e.g. Label Recommendations for Tacrolimus. These recommendations are due, among other things, to the fact that blood levels correlate better with the therapeutic and side effects of drugs than does the dose alone (Shaw et al. 1998) and are useful to identify individual s who are overdosed. Individual overdoses can cause acute or long-term effects such as infections or development of cancers with fatal consequences. For these reasons, TDM is regarded as mandatory in transplantation (Touw et al. 2005).

In the first few months after transplantation, however, there are still usually very few complications due to an individual under- or over-immunosuppression.

A well-known weakness of TDM currently practiced is a results of the fact that immunosuppressive drugs are not analyzed where they actually function, namely within the immune cells; rather, for practical reasons, either in the blood plasma (ie outside cells) or in whole blood. However, the uptake of drugs into the cells and the excretion from cells is very variable for many different reasons (for example genetic variation and interactions with other drugs and diet). There are initial indications from clinical studies (eg, Crettol et al. 2008; Falck et al. 2008) and many other reasons for our assumption that the measurement of intracellular target concentrations of therapeutic immunosuppressive drugs (itc-TDM, intracellular target concentration therapeutic drug monitoring) will allow for much better individual therapy adjustment than does the current practice of measuring concentrations in whole blood or in plasma.

In the long term, our research will help clarify whether the measurement of immunosuppressant concentrations in the target cells can actually lead to an improvement in the therapy. The project carried out here is intended to provide important information as to which intracellular concentrations usually have therapeutic effects and few side effects.

2.3. Current state of therapeutic immune monitoring after liver transplantation

Well-controlled drug concentrations at the target site is only one of many prerequisites for good therapeutic effects. Therefore, the activity of the immune system is measured using laboratory parameters. Three types of partially overlapping parameters or targets can be distinguished:

a) Immuno-activity Markers (Rejection or Early Infection Detection Markers)
b) Immune tolerance markers
c) Additional markers for the early diagnosis and differential diagnosis of abnormal transplant function

Thus, an aim is to identify the signs of under- or over-immune activity as soon as possible prior to any clinically recognizable disfunction using, a) laboratory markers of immunosuppression (early markers of rejection / infection detection) in order to prevent the occurrence of severe infections and rejections. Another aim is to identify with b) immuno-tolerance markers those patients who have good tolerance for the transplanted organ and thus could benefit from a lower dose of immunosuppressants. And in the case of the c) markers for the differential diagnosis of transplant organ function disorders, the aim is to

differentiate between drug toxicity or overdosing and rejection without the need for stressful organ biopsies. In particular, the following parameters will be measured:

Markers of immune activity (early markers of rejection / infection detection)

Cylex® ImmuKnow® - $CD4^+$ immune response. The determination of ATP synthesis in $CD4^+$ T cells after stimulation with phytohemagglutinin serves to estimate the global T cell immune response. The method seems to be suitable for the prediction of infections and possibly also for rejection reactions in patients after organ transplantation (Kowalski et al. 2003; Schulz-Juergensen et al. 2012).

IL-2/$CD8^+$ In individual studies, interleukin-2 (IL-2) secretion in $CD8^+$ T cells was identified after ex vivo stimulation as a predictive marker for transplant rejection (Boleslawski et al. 2004; Millan et al. 2010). In this case, the number of IL-2-expressing cells after secretion inhibition is determined by flow cytometry in relation to the total number of $CD8^+$ T cells.

Immune tolerance markers

B-cell differentiation genes. The investigation of the gene expression of IGKV1D-13, IGKV4-1 and IGLL1 in B cells is a potential method for the identification of operationally tolerant patients and can be carried out by means of quantitative PCR. In a collective of patients after renal transplantation, the expression of these genes differentiated between tolerant and non-tolerant patients (Newell et al. 2010).

FoxP3 expression for the quantification of regulatory T cells (Tregs). The Forkhead Box P3 (Foxp3) is expressed in regulatory T cells (Tregs) whose number appears to correlate with the presence of operational tolerance (i.e., no rejection over 1 year without immunosuppressive medication) in patients after organ transplantation. Currently, the determination of $CD4^+CD25^{high}Foxp3^+$ Tregs in mononuclear cells is being used to quantify Tregs using intracellular fluorescence staining (Pons et al. 2008). In addition, since T cells can also express transient FoxP3, the use of quantification of a FoxP3-specific demethylation was proposed as a surrogate parameter for the determination of Tregs as a way to improve analytical specificity (Wieczorek et al. 2009).

Markers for the differential diagnosis of transplant function disorders

Donor-specific circulating DNA (CNA). Due to genetic differences between the graft donor and recipient, the ratio of circulating donor and recipient DNA in the patient's cell-free plasma may potentially describe both the severity and the course of a rejection reaction. In the case of rejection, the immune reaction leads to apoptosis of donor cells and the DNA released in the process is quantified in the plasma. The sequencing of circulating plasma DNA (CNA) with subsequent quantification represents a potential alternative to complex and invasive biopsies (Snyder et al. 2011).

The latter analyses are not genetic analyses of markers which have specific significance for phenotypes, but the identification of genetic information in these analyzes cannot be prevented so we can make pharmacogenetic analyses as part of the patient information collected.

2.4. Current status of pharmacogenetic testing in immunosuppression

For a number of immunosuppressants, pharmacogenetic typing is increasingly becoming part of everyday medical practice. This is particularly true for the testing of thiopurin S-methyltransferase activity for use of azathioprine (Schütz *et al.* 1995; Compagni *et al.* 2008), testing of CYP3A5 activity for tacrolimus (Kamdem et al., 2005) and possibly variants in the CYP3A4 enzyme (variants **1B*, **22*) for cyclosporin and tacrolimus. Other tests, e.g. variants of the MDR1 appear interesting based on a few studies, but further research studies need to be carried out, since the results are controversial. We would like to comprehensively analyze a very wide range of possible candidate gene variants with respect to the questions posed by the present study using single nucleotide polymorphism analyses (SNP) and re-sequencing.

2.5. Further information about the patient group examined here

Patients should preferably be included in the study as early as possible within the first 2 weeks of liver transplantation (as soon as a correspondingly stable state of health is achieved after the operation or when the patients are able to consent). Inclusion will be considered until the end of the 9th month after transplantation, and the observation period per patient should be 1 year.

In the first weeks and months after transplantation, rejection reactions and infections occur relatively frequently, so that research on innovative biomarkers is of particular interest during this period. A particularly high statistical power results for the question (definition of reference ranges or assessment of a possible benefit) from measurements done in the first few months.

3. Objectives of the study

In the study, patients will be treated as before enrollment. The intracellular concentrations of the immunosuppressants as well as the biomarkers of the immune activity are measured in the context of the present study as will the laboratory tests carried out regularly during the course of their health care, including therapeutic drug monitoring. In addition, circulating DNA differentiated by donor and recipient will be quantified as a measure of organ rejection or cell damage from the immunosuppressants and pharmacogenetic markers will be measured.

The aim is to determine from the analysis of these data, in relation to the clinical data for the patients, which parameters are promising in order to control the selection and dosage of immunosuppressive drugs in the future.

The following specific objectives are to be achieved:

- Correlation of intracellular concentrations in mononuclear blood cells with the concentrations in non-fractionated blood (as usually used for cyclosporin and tacrolimus) or in the plasma (as usually used for mycophenolic acid).
- Determination of therapeutic target intracellular concentrations of tacrolimus, cyclosporin A, mycophenolic acid and everolimus (if approved for immunosuppressive treatment after liver transplantations in Germany) in mononuclear cells in blood by means of the two clinical endpoints infection and rejection and by selected markers of drug toxicity.

- Analysis of the relationship between other side effects of the immunosuppressants used (renal functional impairment, hypercholesterolaemia, diabetogenic effects, diarrhea, leukopenia) in relation to the intracellular drug concentrations (itc-TDM) to answer the question as to whether side effects can be reduced by the subsequent application of itc-TDM.
- Exploration of the diagnostic value of other biomarkers of immune cell activation and immune tolerance in order to identify indicators that allow for early intervention in crises and, on the other hand, allow for minimally effective immunosuppression in order to reduce the side effects of immunosuppression.
- Exploration of the potential diagnostic value of pharmacogenomic biomarkers for the individualization of immunosuppressive therapy. This primarily relates to (1.) variants of importance for tissue concentrations (pharmacokinetics) of immunosuppressants, such as variants in drug membrane transport protein genes (e.g., MDR1) and drug-metabolizing enzymes (e.g., CYP3A4, CYP3A5). This also applies to (2.) gene variants controlling metabolic and signaling pathways which are influenced by the immunosuppressants, and finally (3.) gene variants controlling other proteins which are important for the activity of the immune system.
- The data are intended to provide the basis for statistical models that can be used to make decisions regarding immunosuppressive therapy in the future and explore which of the mentioned biomarkers have the best sensitively and whether combinations of the biomarkers are better than single biomarkers to identify or predict rejections and infections.

As far as our hypotheses are concerned, itc-TDM could result in a reduction in transplant complications and drug side effects, coupled with an improvement in the quality of life and life expectancy of transplant patients. As soon as we have identified the appropriate biomarkers, it seems quite realistic to identify patients for minimization of immunosuppression.

In addition, we would like to collect biomaterial for transplant immunology and transplant immunosuppression to allow for the possibility of evaluating other biomarkers. This particular biobank could be used for research on health progress after solid organ transplants so blood samples and DNA of organ donors should also be stored as long as possible since only the combination of the two can convey a complete picture.

4. Study design

4.1. Primary and secondary endpoints

<u>Primary endpoints</u> are the intracellular immunosuppressant concentrations in mononuclear blood cells (itc-TDM), whose correlation with TDM concentrations measured according to current medical practice will be analyzed.

<u>Secondary endpoints</u> are infection (as a result of over-immunosuppression) and rejection (as a consequence of insufficient immunosuppression).

Infection: Infections will be characterized by infection-type, deterioration of overall health, especially if they result in the administration of systemic anti-infective drugs (antibiotics, antimycotics, and antivirals). Deaths which are most likely to be explained (using clinical data or autopsy) as a result of infections (e.g., sepsis, pneumonia) are also deaths considered to be the result of infections, even if no anti-infective agents have been administered.

Rejection: All deteriorations in the health or function of the transplanted organ which lead to rejection therapy (e.g., highly dosed glucocorticoids or biologicals), will be considered rejections. A death, a loss of graft function, or re-transplantation will also considered to be rejection if, according to the clinical assessments of the physicians and the pathologists involved, rejection was the most likely cause.

Other secondary clinical endpoints will include all clinical and laboratory parameters that may provide evidence of under or over-immunosuppression or other forms of drug side effects. In doing so, fundamentally, recording will be limited to the minimum necessary and only those parameters will be included in the study documents and evaluation which are recorded during the scope of routine clinical care (i.e. not research-related).

The following table lists all parameters. Those that will contribute to the evaluation of the primary endpoints are highlighted in bold.

Parameter		Reason
**itc-TDM**		**Primary endpoint**
gGT	Gamma-Glutamyltransferase	Rejection, drug hepatotoxicity (inter alia, cholestasis)
ALAT	Alanin-Aminotransferase	Rejection, drug caused hepatotoxicity
ASAT	Aspartat-Aminotransferase	Rejection, drug caused hepatotoxicity
AP	Alkaline Phosphatase	Rejection, drug caused hepatotoxicity (cholestasis)
Bilirubin	Total-Bilirubin	Liver function
Albumin		Liver function
INR	*International normalized ratio* (normierte Prothrombinzeit)	Liver function
Cr	Serum-Creatinine Concentration	Renal function, drug caused nephrotoxicity
Leukocytes	Total number of leukocytes in blood	Infection, drug cause hepatotoxicity
Lymphocytes	Total number of lymphocytes in blood	Infection, drug cause hepatotoxicity
HbA1C	Glycosylated Hemoglobin	Drug toxicity
Total cholesterol		Drug toxicity
LDL-Cholesterol		Drug toxicity (uptake inhibited by Cyclosporin)
Triglycerides		Drug toxicity (VLDL, Insulin-Signaling)
Detection of the side effects of immunosuppressive medication	Clinical notes during clinic visits	Drug toxicity
Blood pressure	Clinic measurements	Drug toxicity
Infections	Clinical notes during clinic visits	Secondary endpoint
Rejection	Biopsy	Secondary endpoint
Total Hospitalizations	Total hospital days	Life quality measure
Immunosuppressive drug	Hospital stays caused by	

related Hospitalizations	complications of immunosuppression	

4.2. Study design

This is a prospective non-interventional observational study (cohort study). All therapies used at the respective centers will be performed clinically in the same way as before the study. The relevant details of the therapies will be documented.

Blood tests will be carried out at the time points shown below only at times when control examinations involving venous puncture or other blood sampling are also carried out outside of this observational study.

4.3. Measures to guard against systematic errors or distortions of the results

The inclusion and exclusion criteria shall be kept as broad as possible to provide the highest possible representativeness (external validity).

Additional innovative biomarkers may be analyzed using the blood samples taken during routine therapeutic care The predicted value of these biomarkers with regard to primary and secondary endpoints should be analyzed. To avoid any interference in routine care, any non-clinically indicated, additional analyzed pharmacological and immunological and / or cell biologic markers will neither be provided to those responsible for the patient’s care nor made available to the personnel responsible for the documentation of the clinical data (principle of blinding).

4.4. Examination of biomarkers

The evaluation will be based on all clinical markers and therapeutic drug monitoring currently being routinely used clinically, as well as the innovative biomarkers which will be specifically explored here. In the following table, these biomarkers are listed with a brief justification for each. Further background information can be found in the introductory chapters. Overall, the additional, scientifically justified blood samples collected will involve only minimal stress and only minimal risk for the patients, since these blood samples will only be collected during the course of other, medically indicated control examinations and since an additional withdrawal of up to 20 ml blood neither subjectively nor sensitive objectively measured leads to physiological changes.

Parameters (abbreviations, parameters, units)		Justification and any further explanations	Necessary blood collection as required for research, as part of a clinically indicated visit
	Cyclosporine in Blood	Transferred from routine clinical measurements into the data forms	0 (since occurring anyway)
	Tacrolimus in Blood	Transferred from routine clinical measurements into the data forms	0 (since occurring anyway)
MPA	Mycophenolic acid in Plasma	If performed for routine clinical examinations and results transferred into the	0 (since occurring anyway)

		data	
	(Everolimus* in Blood)	If performed for routine clinical measurements, results transferred into the data evaluations	0 (since occurring anyway)
Itc-CyA	Intracellular Cyclosporine	Primary biomarker to be investigated for cyclosporine therapy	6 ml
Itc-Tacro	Intracellular Tacrolimus	Primary biomarker to be investigated for Tacrolimus therapy	
Itc-MPA	Intracellular Mycophenolic acid	Primary biomarker to be investigated for MPA therapy	
(Itc-Everolimus*)	(Intracellular Everolimus)	Primary biomarker to be investigated for Everolimus therapy	
CD4+ Immun-antwort	ng/ml ATP in PHA-stimulated CD4+ T-Cells	Immune activity marker	3 ml
IL-2/CD8+	Proportion of IL-2+ T-Cells of total CD8+ T-Cells, measured as percent	Immune activity marker	3 ml
IGKV1D-13, IGKV4-1 und IGLL1	Expression of tolerance-relevant B-cell differentiation genes, normalized RNA copy number	Immune tolerance marker	3 ml
FoxP3	As FACS-Measurement	Immune tolerance marker	3 ml
FoxP3	As DNA-Methylation Measurement	Immune tolerance marker	2 ml
CNA	DNA circulating in plasma	Marker for differential diagnosis of rejection versus other organ damage	0 (from supernatant after cell isolation)
			Total of 20 ml Blood for research testing

Items marked with * will be done only after approval for immunosuppressive therapy in Germany

Pharmacogenetic biomarkers

<u>Patients' DNA</u> will be used for screening both candidate gene-related and a hypothesis-free screening gene variant with significance for drug effects or side effects as well as genes having relevance for rejection or infection risk will be investigated.

<u>DNA from the transplanted organ</u> will be used for screening both candidate gene-related as well as a hypothesis-free screening gene variants with significance for drug effects or side effects as well as genes having significance for rejection risk will be investigated.

In some cases (unclear side-effects or poorly explained rejections), a blood concentration measured 2 hours after dosing (C2) or a blood concentration profile for the medicinal products used may be determined in individual patients. If such measurements are carried out in patients included in the study, intracellular concentrations will also be recorded.

4.5. Study duration and schedule

The study as a whole should begin as soon as possible. After the starting the study should take 18 months. The observation period is 1 year any individual patient.

For an individual patient, the study visit times were chosen based on usual, expected clinic visits and hospital stays. In addition, there are additional visits for possible complications (e.g., rejection or infection). Both, the regular visits as part of the follow-up and the visits or hospitalizations for complications will not occur for the study. Additionally, the study schedule (see below) only serves as a guide to the maximum number of additional laboratory analyses.

Nr.	Time post LTx*	Range post LTx*	TDM (Blood, Plasma)**	Itc-TDM (intracellular)	CD4- Immune response	IL-2/CD8	FoxP3	B-Zell- Differentiation genes	CNA (circulating DNA)	Clin-Chem Analysis**	Clinical Data**	Recording of Events**	Pharmacogenetics
0		Once between											X
1	1 Week	0 – 10 Days	X	X		X	X	X	X	X	X	X	
2	2 Weeks	11 – 30 Days	X	X	X	X	X	X	X	X	X	X	
3	6 Weeks	1 – 2 Months	X	X		X	X	X	X	X	X	X	
4	3 Months	2 – 4 Months	X	X		X	X	X	X	X	X	X	
5	6 Months	4 – 8 Months	X	X	X	X	X	X	X	X	X	X	
6	9 Months	8 – 10 Months	X	X		X	X	X	X	X	X	X	
7	12 Months	10 – 14 Months	X	X	X	X	X	X	X	X	X	X	
With respect to acute complications (suspected or confirmed acute rejections and / or infections)***			x	x	X	X			X	X	X	X	
Based on documentation***			x	x	X	X			X	X	X	X	

* None of the mentioned dates are applicable for patients who do not enter ambulatory aftercare during this period.

** These measurements or surveys will be evaluated in the course of this observational study, but will not be done only for the study.

*** These measurements will be performed to better estimate the parameters. We are aware that biomarkers measured during acute events can be both, the cause and the result of an event.

Therefore alterations of a biomarker measured during acute events do not necessarily mean that this biomarker would have any predictive value.

4.6. Cancellation criteria

This observational study is associated with only minimal risks, so there are no abortive criteria for investigating any individual participant or the study as a whole. The independent reasons for terminating the study for individual patients are shown below. Stopping the entire study or only one of the study centers for financial or organizational reasons is very unlikely, but can not be completely excluded.

4.7. Data management

All data from this study will be recorded on paper in GCP-compatible forms or entered into correspondingly validated databases. For data entered into the Microsoft Excel program, which is not known to be GCP-compliant, the corresponding data sheets will be printed out and printed in the subject’s study folder.

A GCP-compliant database will be created in which all data will be anonymized.

5. Selection and exclusion of study participants

5.1. Inclusion criteria

Consented patients within the first 24 months after a liver transplantation.

5.2. Exclusion criteria

Any participation in therapy examinations for non-included immunosuppressive drugs, unless the participation in the present observational study has been approved in writing by the respective sponsor.

Lack of consent
Age <18 years

5.3. Criteria for individual study termination and documentation in such cases

- Termination at the request of the individual patient (if possible, reasons should be recorded)
- Withdrawal of consent to continue in the study by a patient
- Termination due to necessity for a re-transplant
- Decision of the study center to include the patient in a research study involving a new immunosuppressive drug

6. Drug therapy and further treatment

The study will have no effect on the medications taken. During the observation period all immunosuppressive medications will be documented as completely as possible.

Similarly, any anti-infective medications (antibiotics, anti-viral medications, antimycotics) and antihypertensive, anti-diabetic and cholesterol-lowering therapies will be documented for evaluation of secondary endpoints.

7. Statistics

7.1. Statistical data evaluation

First, all measured biomarker values will be presented as descriptive statistics for the entire sample and for the subgroups with rejection within 2 weeks after a measurement or infection within 2 weeks after a measurement. Further evaluation of the biomarkers will be based on multivariate mixed model analyses, based on the many years of experience in the Department of Medical Statistics (Director: Prof. Dr. Tim Friede). In addition, pharmacokinetic-pharmacodynamic model analyses will be conducted with the aim of exploring which biomarker-based calculated dosage recommendations might be expected in the future. These are of course not intended to be used in the present study, but in the long-term perspective these could certainly be part of a personalized medicine approach.

7.2. Sample size justification

We plan to carry out the study on 125 patients who can be tracked for more than one year. We assume that about 20% will leave the study too early for different reasons and will not be evaluable (drop out) so we will try to include 150 patients initially.

The analysis of the intracellular target concentrations of the drugs will be based on the correlation of the intracellular target concentrations with the currently measured total blood concentrations. A sample size of 115 patients would be sufficient to achieve a correlation coefficient (Pearson) of at least 0.3 with a sufficiently precise determination of intracellular target concentrations.

Assuming an incidence of infections and rejections in the first year after transplantation of approximately 30% there will be about 40 rejections and about 40 infections among the remaining 125 patients. As a result, the number of patients provided is likely to be sufficient to estimate the therapeutic suitability of the different biomarkers based on these clinical endpoints.

Any further analyzes are regarded as secondary and will be evaluated in the context of the actual cases.

7.3. Criteria for discontinuation of studies

Listing of all medical or scientific reasons which would lead to study termination is not feasible. Therefore, there are no predefined criteria for study discontinuation.

7.4. Handling of missing and incomplete data

We believe that very few patients will discontinue participation in the study, as there will not be any significant additional stress from study participation, or medical controls that will be necessary regardless of the study. Despite this, the multivariate mixed models to be used in the evaluation are quite robust against missing values (Molenberghs G, Kenward MG (2007) Missing Data in Clinical Studies; Chapter 7). If high proportions of dropouts would occur, we could also investigate the hypothesis of independent dropouts using the above-mentioned methods.

7.5. Changes to the planned statistical evaluation

All changes will be documented, justified and communicated as an administrative amendment to the responsible Ethics Committee(s).

7.6. Criteria for exclusion of data and patients from statistical evaluation and publication

Measurements whose values deviate more than 3 standard deviations upwards or downwards from the expected value will be repeated if possible. Exclusion of values in the analysis will occur only in cases where, according to a written and reasoned assessment by two experts, the measurement is medically highly unlikely. Not evaluated patients will be replaced by another patient as will patients with less than 3 measurements, unless caused by a clinical endpoint (infection or rejection) and the patient cannot be followed longer in the study, Patients who either die, require a re-transplant or a basically different immunosuppressive regimen, or who no longer meet the inclusion criteria will be terminated from the study.

8. Access to source data

In terms of data quality, the study centers and study patients will agree to the examination by study staff of the original data. Only authorized study staff will have the right to receive training to do this, and will be known to the respective centers through the use of a study delegation list. Participating institutions will demonstrate the ability to handle and document patient data in a confidential way and will understand and sign a data protection declaration. Patients will also agree that in case of questions related to this study, any questions or required information should be requested from their attending physicians.

9. Measures to ensure data quality

Regular monitoring will be carried out at all study centers. A monitoring plan has been created for this purpose. All data will be documented in the original documents and entered in a validated database with an audit trail.

10. Ethical and legal aspects

In the course of this study, no study initiated changes in drug therapy will be made. Blood samples used to measure the additional innovative biomarkers of interest will only be carried out as part of regular monitoring, so no additional venipunctures are required.

Per visit, 20 ml of blood will be collected for the measurement of the molecular biomarkers. This will generally occur at intervals of greater than one month. No separate, study only related venipunctures will be performed. The additional amounts of blood are felt to be associated with minimal risk and only minimal extra burden.

A certain degree of inconvenience of study participants is required for data collection but is felt to be balanced by the potential for significantly improving future therapy for this group of patients. The patients themselves might also benefit from the knowledge gained.

This is a non-interventional study. The aim of the study is not to study the metabolism, pharmacokinetics, therapeutic or side effects of the drugs, since all these facts are well known. However, these data are needed to evaluate possibly clinically valuable new methods of therapeutic drug monitoring.

With regard to the Medical Devices Act, no new in vitro diagnostic tests will be used for clinical decisions. Of course only certified products will be used for blood sampling. In the long term it is conceivable that, depending on the success of the data collected here, that we will be able to develop novel in vitro diagnostics to facilitate practical care. However, the development of new in vitro diagnostics is not planned for the present study. All laboratory analyzes are carried out laboratories which are certified to conduct clinical-chemical laboratory diagnostic testing in a GCP-compliant manner.

11. Data management

All data will be documented in paper form and stored in a GCP-compatible database.

12. Study Financing and insurance

The study is funded by the Federal Ministry of Education and Research (funding ID code 01ES1102).

As the study is only conducted within the context of clinical visits and blood tests, the health risks are minimal. No patient insurance is included.

13. Publication agreements

All scientific data from the study will be published after completing the planned number of cases.

All questions regarding publications will be discussed by a publication committee in person or by circulated e-mails. The publication committee is composed of at least the following persons: at least one treating physician and Professors Oellerich, Friede and Brockmöller. Additional persons may be included in the publication committee.

Individual publications of individual study aspects before the respective final publication as well as the publication of doctoral theses will also be supported by the provision of corresponding data from the database and from the laboratory test results.

In the case of doctoral theses; however, it should be clarified with the doctoral candidate(s) that the publication of their PhD thesis may have to be delayed until after scientific publication.

14. Supporting Literature

Boleslawski, E., F. Conti, et al. (2004). "Defective inhibition of peripheral CD8+ T cell IL-2 production by anti-calcineurin drugs during acute liver allograft rejection." Transplantation **77**(12): 1815-20.

Compagni, A., S. Bartoli, et al. (2008). "Avoiding adverse drug reactions by pharmacogenetic testing: a systematic review of the economic evidence in the case of TPMT and AZA-induced side effects." Int J Technol Assess Health Care **24**(3): 294-302.

Crettol, S., J. P. Venetz, et al. (2008). "Influence of ABCB1 genetic polymorphisms on cyclosporine intracellular concentration in transplant recipients." Pharmacogenet Genomics **18**(4): 307-15.

Falck, P., A. Asberg, et al. (2008). "Declining intracellular T-lymphocyte concentration of cyclosporine a precedes acute rejection in kidney transplant recipients." Transplantation **85**(2): 179-84.

Holt, D. W., V. W. Armstrong, et al. (2002). "International Federation of Clinical Chemistry/International Association of Therapeutic Drug Monitoring and Clinical Toxicology working group on immunosuppressive drug monitoring." Ther Drug Monit **24**(1): 59-67.

Kamdem, L. K., F. Streit, et al. (2005). "Contribution of CYP3A5 to the in vitro hepatic clearance of tacrolimus." Clin Chem **51**(8): 1374-81.

Kowalski, R., D. Post, et al. (2003). "Immune cell function testing: an adjunct to therapeutic drug monitoring in transplant patient management." Clin Transplant **17**(2): 77-88.

Millan, O., C. Benitez, et al. (2010). "Biomarkers of immunoregulatory status in stable liver transplant recipients undergoing weaning of immunosuppressive therapy." Clin Immunol **137**(3): 337-46.

Newell, K. A., A. Asare, et al. (2010). "Identification of a B cell signature associated with renal transplant tolerance in humans." J Clin Invest **120**(6): 1836-47.

Pons, J. A., B. Revilla-Nuin, et al. (2008). "FoxP3 in peripheral blood is associated with operational tolerance in liver transplant patients during immunosuppression withdrawal." Transplantation **86**(10): 1370-8.

Schulz-Juergensen, S., M. M. Burdelski, et al. (2012). "Intracellular ATP production in CD4+ T cells as a predictor for infection and allograft rejection in trough-level guided pediatric liver transplant recipients under calcineurin-inhibitor therapy." Ther Drug Monit **34**(1): 4-10.

Schütz, E., J. Gummert, et al. (1995). "Azathioprine myelotoxicity related to elevated 6-thioguanine nucleotides in heart transplantation." Transplant Proc **27**(1): 1298-300.

Shaw, L. M., B. Kaplan, et al. (1998). "Prospective investigations of concentration-clinical response for immunosuppressive drugs provide the scientific basis for therapeutic drug monitoring." Clin Chem **44**(2): 381-7.

Snyder, T. M., K. K. Khush, et al. (2011). "Universal noninvasive detection of solid organ transplant rejection." Proc Natl Acad Sci U S A **108**(15): 6229-34.

Touw, D. J., C. Neef, et al. (2005). "Cost-effectiveness of therapeutic drug monitoring: a systematic review." Ther Drug Monit **27**(1): 10-7.

Wieczorek, G., A. Asemissen, et al. (2009). "Quantitative DNA methylation analysis of FOXP3 as a new method for counting regulatory T cells in peripheral blood and solid tissue." Cancer Res **69**(2): 599-608.
