# Supplementary material for: Graft-derived cell-free DNA, a noninvasive early rejection and graft damage marker in liver transplantation: A prospective, observational, multicenter cohort study
Source: PLoS Med. 2017 Apr 25;14(4):e1002286. doi: 10.1371/journal.pmed.1002286 (PMC5404754; doi:10.1371/journal.pmed.1002286)
Supplement: S3 Text — (PDF) [file pmed.1002286.s016.pdf]

09 Oct. 2012

Prospective analysis plan

Charité/Berlin, UKE/Hamburg-Eppendorf, UMG/Göttingen

**Visit**	**1**	**2**	**3**	**4**	**5**	**6**	**7**	**VU**
**Time after LTx**	**0-10 days**	**11-30 days**	**1-2 months**	**2-4 months**	**4-8 months**	**8-10 months**	**10-14 months**	**clinical events**
**GcfDNA**	x	x	x	x	x	x	x	x
LFTs and ISD drug concentrations will be measured as per local laboratory routine analyses and all biopsies will be performed, analyzed and reported as per standard of care at the three different transplant centers.								

Additional GcfDNA testing in a subset of patients (n=25) from UMG/Göttingen

**Time after LTx**	**15 min**	**6 h**	**1 day**	**2 days**	**3 days**	**4 day**	**5 days**	**6 days**	**7 days**	**8 days**	**9 days**	**10 days**	**11 days**	**12 days**	**13 days**	**14 days**	**17 days**	**20 days**	**23 days**	**26 days**	**29 days**
**GcfDNA**	x	x	x	x	x	x	x	x	x	x	x	x	X	x	x	x	x	x	x	x	x
Blood samples will be drawn during the first month post LTx (or until hospital discharge if earlier). LFTs and ISD drug concentrations will be measured as per local laboratory routine analyses and all biopsies will be performed, analyzed and reported as per standard of care at the UMG/Göttingen transplant center.																					

(ISD, immunosuppressant drug; LFTs, liver function tests; LTx, liver transplantation)
